# Ending malnutrition in all its forms requires scaling up proven nutrition interventions and much more: a 129-country analysis

**DOI:** 10.1186/s12916-020-01786-5

**Published:** 2020-11-13

**Authors:** Nick Scott, Dominic Delport, Samuel Hainsworth, Ruth Pearson, Christopher Morgan, Shan Huang, Jonathan K. Akuoku, Ellen Piwoz, Meera Shekar, Carol Levin, Mike Toole, Caroline SE Homer

**Affiliations:** 1 Maternal, Child and Adolescent Health Program, Burnet Institute, Melbourne, Australia; 2 School of Public Health and Preventative Medicine, Monash University, Melbourne, Australia; 3 School of Population and Global Health, University of Melbourne, Melbourne, Australia; 4 Jhpiego, Baltimore, MD USA; 5 World Bank Group, Washington DC, USA; 6 Nutrition Global Development Program, Bill and Melinda Gates Foundation, Seattle, USA; 7 Department of Global Health, University of Washington, Seattle, USA

**Keywords:** Economic analysis, Mathematical model, Nutrition, Optima Nutrition, Sustainable Development Goals

## Abstract

**Background:**

Sustainable Development Goal (SDG) 2.2 calls for an end to all forms of malnutrition, with 2025 targets of a 40% reduction in stunting (relative to 2012), for wasting to occur in less than 5% of children, and for a 50% reduction in anaemia in women (15–49 years). We assessed the likelihood of countries reaching these targets by scaling up proven interventions and identified priority interventions, based on cost-effectiveness.

**Methods:**

For 129 countries, the Optima Nutrition model was used to compare 2019–2030 nutrition outcomes between a status quo (maintained intervention coverage) scenario and a scenario where outcome-specific interventions were scaled up to 95% coverage over 5 years. The average cost-effectiveness of each intervention was calculated as it was added to an expanding package of interventions.

**Results:**

Of the 129 countries modelled, 46 (36%), 66 (51%) and 0 (0%) were on track to achieve the stunting, wasting and anaemia targets respectively. Scaling up 18 nutrition interventions increased the number of countries reaching the SDG 2.2 targets to 50 (39%), 83 (64%) and 7 (5%) respectively. Intermittent preventative treatment of malaria during pregnancy (IPTp), infant and young child feeding education, vitamin A supplementation and lipid-based nutrition supplements for children produced 88% of the total impact on stunting, with average costs per case averted of US$103, US$267, US$556 and US$1795 when interventions were consecutively scaled up, respectively. Vitamin A supplementation and cash transfers produced 100% of the total global impact on *prevention* of wasting, with average costs per case averted of US$1989 and US$19,427, respectively. IPTp, iron and folic acid supplementation for non-pregnant women, and multiple micronutrient supplementation for pregnant women produced 85% of the total impact on anaemia prevalence, with average costs per case averted of US$9, US$35 and US$47, respectively.

**Conclusions:**

Prioritising nutrition investment to the most cost-effective interventions within the country context can maximise the impact of funding. A greater focus on complementing nutrition-specific interventions with nutrition-sensitive ones that address the social determinants of health is critical to reach the SDG targets.

## Background

Undernutrition contributes to an estimated 45% of child deaths globally [1]. Stunting (height-for-age more than two standard deviations below the World Health Organization (WHO) Child Growth Reference Standards median) and wasting (weight-for-height more than two standard deviations below the WHO Child Growth Standards median) are associated with higher risk of severe illness and death. Other forms of undernutrition affect child brain development, impairing learning and future earning capacity [2]. Together, these conditions increase child mortality, exacerbate poverty, create entrenched intergenerational disadvantage and hinder the economic future of a country. The Sustainable Development Goals (SDGs) target 2.2 calls for ending malnutrition in all its forms [3]. Progress is tracked by four nutritional indicators; stunting and wasting levels in children under 5 years of age, anaemia in women of reproductive age (15–49 years) and overweight among children under five. There is an aspiration to achieve, by 2025, a 40% relative reduction in stunting (to a global prevalence of approximately 15%), a reduction in child wasting to < 5% prevalence, and a 50% relative reduction in anaemia in women (to a global prevalence of approximately 15%), with 2012 as the baseline year. [3]. Several analyses have shown that despite improvements in child stunting and wasting over the last two decades [4], most countries are not on track to reach the SDG 2.2 undernutrition targets [5].

Today there exists a suite of proven nutrition interventions that address the immediate causes of undernutrition, widely referred to as “nutrition-specific interventions”. It is well-established that non-nutritional factors affect nutritional status, including subclinical inflammation and untreated infections, environmental pathogen exposure, gender inequality and women’s lack of agency and empowerment. Policies and interventions to address these underlying factors, such as health systems strengthening, improved water, sanitation and hygiene (WASH) and women’s empowerment, are called “nutrition-sensitive” because they address underlying social, environmental and health-related nutrition determinants, and improvements in nutritional status may result when these interventions are implemented at scale (as illustrated in UNICEF’s conceptual framework [6]).

Evidence for the impact of nutrition-specific interventions comes from systematic reviews and meta-analyses of findings from randomised controlled efficacy and effectiveness studies that are summarised in the WHO guidelines [7, 8] and multiple Lancet nutrition series (2008 [9], 2013 [10], 2019 [11]). The cost and marginal impact of policies and interventions to address underlying determinants is less amenable to controlled study, and information about their role is primarily based on quantitative decomposition analyses of cross-sectional data collected over time [12–21]. This asymmetry in the evidence base makes it difficult to model and compare the cost-effectiveness of these different types of interventions or to measure their complementary impacts; however, in recent years, the number of studies to address this evidence gap is increasing. An additional challenge is that data on coverage of most proven nutrition interventions is lacking, making it difficult to assess baselines and progress towards implementing interventions [22].

Previous work has estimated the cost and impact of scaling up evidence-based nutrition interventions. In 2010, Horton and colleagues [23] estimated that it would cost US$10.3 billion per annum globally to scale up the 13 nutrition interventions from the 2008 Lancet series [9], and that doing so could reduce stunting by 20% and the prevalence of severe acute malnutrition by 50%. In 2013, Bhutta and colleagues [10] estimated that it would cost US$9.6 billion per annum to scale up 10 nutrition-specific interventions in 34 countries (90% of the world’s stunting burden), and that this could reduce stunting by 20% and the prevalence of severe wasting by 61%. In 2017, Shekar and colleagues [24] provided an investment framework for reaching the SDG 2.2 undernutrition targets (and the global nutrition target of 50% of infants < 6 months exclusively breastfed by 2025 [25]). They estimated that the world needs US$70 billion over 10 years to invest in high-impact nutrition-specific interventions in order to reach these targets, combined with continued improvements in WASH and other underlying determinants [26].

Investment cases and costing estimates for nutrition interventions are vital for their adoption; however, budget constraints mean that countries will not be able to scale up all available nutrition interventions. Decisions must be made about how to prioritise interventions, and countries will benefit from knowing what the most cost-effective strategies are to maximise the impact of limited funds. Optima Nutrition [27] is a mathematical model that estimates the impact of scaling up combinations of 18 different nutrition interventions on stunting, wasting and mortality in children under five and anaemia in women of reproductive age. These interventions are included in the model based on systematic reviews, meta-analyses and the findings of multiple Lancet series; each intervention has been shown in isolation to lead to improved nutrition outcomes. The model also includes an economic component and can generate average cost-effectiveness estimates for interventions.

This first aim of this study was to expand the scope of previous analyses by estimating the progress that could be made towards the SDG 2.2 undernutrition targets by scaling up 18 nutrition interventions (for which there is currently evidence of effectiveness) within 129 individual low- and middle-income countries (LMICs). The second aim was to estimate which interventions countries should prioritise, to provide countries guidance on how to maximise impact when investment is limited.

## Methods

### The Optima Nutrition model

The Optima Nutrition model tracks the number of women of reproductive age (15–49 years) in a population, who can become pregnant and give birth. After birth, children are tracked until 5 years of age across five age bands and are categorised according to their mother’s breastfeeding practices, family economic status, height-for-age (stunting) status, weight-for-height (wasting) status and anaemia status (Additional file 1: Fig. A1) [27–49]. Children in the model can die from a range of specific causes, with the relative risks of dying from each cause related to the child’s breastfeeding, stunting, wasting and anaemia status according to global published estimates.

Several risk factors for stunting in children are modelled: birth outcomes (preterm birth and/or a child being born small for gestational age [SGA]), stunting at a younger age band, sub-optimal feeding practices (age-appropriate breastfeeding and complementary foods) and incidence of diarrhoea (Additional file 1: Fig. A2). In addition, anaemia in women of reproductive age is modelled to be a risk factor for sub-optimal birth outcomes, birth outcomes and diarrhoea incidence are modelled to be risk factors for wasting and sub-optimal breastfeeding is modelled to be a risk factor for diarrhoea incidence.

In the model, interventions can improve nutritional outcomes directly or indirectly by reducing risk factors. For example, changes to breastfeeding practices can reduce diarrhoea incidence which indirectly reduces stunting. Changing the coverage of an intervention among its target population leads to changes in projected nutrition outcomes based global estimates of intervention effectiveness (Additional file 1: Fig. A2). The cost of achieving a given intervention coverage is calculated by multiplying the number of beneficiaries by the intervention’s estimated unit cost per beneficiary (see below).

Eighteen nutrition interventions were considered in this study based on the available literature and global recommendations (Tables 1, 2, and 3). These include various nutrition supplements for pregnant women, micronutrient supplements for children under five, treatment of severe acute malnutrition for children, lipid-based nutrition supplements for children 6–23 months at risk of food insecurity and/or poor growth, treatment of diarrhoea for children, nutrition education and interventions for reducing malaria.

**Table 1:** Intervention target populations and effects

Target populations and effects			
Intervention	Target population	Effects	Source / Effect sizes
Cash transfers (unconditional)	Children below the poverty line	Reduces the incidence of SAM Reduces the incidence of MAM	RRR = 0.32 (0.16-0.61) for SAM incidence RRR = 0.40 (0.23-0.68) for MAM incidence [Langendorf et al. 2014, PLoS Med [50], Niger study comparing super cereal plus + cash (US$52 per month) compared to super cereal plus.]
Delayed umbilical cord clamping	Pregnant women (at birth, but impact is for children <1 month)	Reduces anaemia	RRR = 0.53 (0.40-0.70) for being anaemic [Hutton and Hassan, 2007 Jama [51]]
Infant and young child feeding (IYCF) education	**Home/community promotion for children 0-23 months:**		
	For children < 1 months	Increases exclusive breastfeeding	OR = 2.17 (1.84-2.56) for exclusive breastfeeding [Sinha et al. 2017 J Nutr [52] for interventions delivered in home or community settings in low- and middle-income countries]
	For children < 6 months	Increases exclusive breastfeeding	OR = 2.48 (1.99-3.09) for exclusive breastfeeding [Sinha et al. 2017 J Nutr [52] for interventions delivered in home or community settings in low- and middle-income countries]
	For children 6-23 months	Increases age-appropriate (partial) breastfeeding	OR = 1.82 (1.36-2.45) for age-appropriate breastfeeding; [Sinha et al. 2017 J Nutr [52]]
	For children 6-23 months	Promotion of appropriate complementary feeding reduces odds of stunting	OR = 0.77 for stunting; [Panjwani et al. 2017 J Nutr [53] food secure population with nutrition education or counselling compared to receiving no intervention]
Immediate initiation of breastfeeding	Children < 1 month	Increases exclusive breastfeeding Reduces deaths due to prematurity	OR = 1.50 (1.26-1.78) for exclusive breastfeeding in children < 1 month. OR = 1.39 (1.11-1.74) for exclusive breastfeeding in children 1-6 months [Boundy et al. 2016, Pediatrics [54]] RRR = 0.49 (0.29-0.82) for mortality due to prematurity [Lawn et al. 2010, I J Emi 2010 [55]]
Lipid-based nutrition supplements	Children 6-23 months old who live in households below the poverty line	Reduces the odds of stunting Reduces the incidence of SAM Reduces the incidence of MAM Reduces anaemia	OR = 0.89 for stunting [Panjwani et al. 2017 J Nutr [53] food insecure with supplementation compared to no supplementation] RRR = 0.915 for SAM and MAM incidence [based on Panjwani et al. 2017 J Nutr [53] food insecure with supplementation compared to no supplementation] RRR = 0.69 (0.60-0.78) for anaemia [De-Regil et al. 2013 Cochrane review [56], assumed the same as micronutrient powders]
Oral rehydration solution (ORS) + Zinc	Children 0-59 months (different quantity by age)	Reduces diarrhoea mortality	RRR = 0.24 (0.15-0.38) for diarrhoea mortality. Calculated as RRR = 0.31 (0.20-0.49) for ORS [Munos, et al. 2010, I J Epi [57]], with additional RRR of 0.77 due to the addition of zinc [Walker & Black 2010, I J Epi [58]]
Public provision of complementary foods	Children 6-23 months old who live in households below the poverty line	Reduces the odds of stunting Reduces the incidence of SAM Reduces the incidence of MAM	OR = 0.89 for stunting [Panjwani et al. 2017 J Nutr [53] food insecure with supplementation compared to no supplementation] RRR = 0.915 for SAM and MAM incidence [based on Panjwani et al. 2017 J Nutr [53] food insecure with supplementation compared to no supplementation]
Treatment of severe acute malnutrition (SAM)	Children experiencing SAM	Increases recovery from episode	78% recovery for wasting among children receiving intervention [Bhutta et al. 2013, Lenters et al. 2013 [10, 59]]. Note that this intervention is defined as treating children until they reach a weight-for-height of three standard deviations below the WHO Child Growth Standards median, at which point their mortality risks are significantly reduced but they are still defined as being wasted (i.e. children who are severely wasted are treated to become only moderately wasted, but wasted nonetheless).
Vitamin A supplementation	Children 6-59 months	Reduces diarrhoea incidence mortality	RRR = 0.85 (0.82-0.87) for diarrhoea incidence [Imdad et al. 2017, Cochrane review [60]] RRR = 0.88 (0.79-0.98) for diarrhoea-specific mortality [Imdad et al. 2017, Cochrane review [60]]
Balanced energy-protein supplementation	Pregnant women below the poverty line	Reduces risk of small for gestational age (SGA) birth outcomes	RRR = 0.79 (0.69-0.90) for SGA birth outcomes [Ota et al. 2015, The Cochrane Library [61]]
Calcium supplementation	Pregnant women	Reduces maternal mortality (hypertensive disorders) Reduces pre-term births	RRR = 0.80 (0.66-0.98) for maternal mortality [Hofmeyr et al. 2018 Cochrane review [62]] RRR = 0.76 (0.60-0.97) for preterm birth [Hofmeyr et al. 2018 Cochrane review [62]]
Iron and folic acid supplementation	Women of reproductive age (pregnant / non-pregnant)	Reduces anaemia Reduces neonatal mortality	RRR = 0.33 (0.16-0.69) for anaemia in pregnant women [Pena-Rosas et al, Cochrane Database Reviews 2015 [63]] RRR = 0.73 (0.56-0.95) for anaemia in non-pregnant women [Fernandez-Gaxiola & De-Regil 2011, Cochrane Database Syst Rev [64]]
Intermittent preventative treatment of malaria during pregnancy	Pregnant women in areas where there is malaria risk	Reduces anaemia Reduces SGA birth outcomes	RRR = 0.83 (0.74-0.93) for being anaemic [Radeva-Petrova et al. 2014, The Cochrane Library [65]] RRR = 0.65 (0.55-0.77) for SGA birth outcomes [Eisele et al. 2010, I J Epi [66]]
Multiple micronutrient supplementation	Pregnant women	Reduces anaemia and risk of SGA birth outcomes	RRR = 0.33 (0.16-0.69) for anaemia in pregnant women [Pena-Rosas et al, Cochrane Database Reviews 2015 [63]] RRR = 0.92 (0.88-0.97) for SGA births [Keats et al. 2019 Cochrane Database Reviews [67]]
Iron and folic acid fortification (wheat, maize or rice)	Everyone	Reduces anaemia Reduces neonatal mortality	OR = 0.976 (0.975-0.978) for being anaemic [Barkley et al. 2015, B J Nutrition [68]] RRR = 0.87 (0.84-0.89) of neonatal mortality [prevention of neural tube defects Blencowe et al. 2010, I J Epidemiology [69]]
Iron and iodine fortification of salt	Everyone	Reduces anaemia Reduces neonatal mortality	OR = 0.976 (0.975-0.978) for being anaemic [Barkley et al. 2015, B J Nutrition [68]]
Long-lasting insecticide-treated bed nets	Everyone in areas where there is malaria risk	Reduces anaemia Reduces SGA birth outcomes	RRR = 0.83 (0.74-0.93) for anaemia [Radeva-Petrova et al. 2014, The Cochrane Library [65]] RRR = 0.65 (0.55-0.77) for SGA birth outcomes [Eisele et al. 2010, Int J Epi [66]]

**Table 2:** Estimated 2018 intervention coverage.

Coverage			
Intervention	*Global average*	*[Between countries: median; inter-quartile range (IQR) and range between countries]* *Low income country (LC), lower-middle income country (LMC) and upper-middle income country (UMC) averages*	Sources and notes
Cash transfers (unconditional)	0%		Not available; set to 0% at baseline.
Delayed umbilical cord clamping	0%		Not available; set to 0% at baseline.
Infant and young child feeding education	30.3%	[Between countries: median=26%; IQR=0-49.4%; range=0-89.6%] LC average=22.6%, LMC average=38%, UMC average=28.4%	LiST^a^ [34]. The target population for IYCF education was taken to be all children 0-23 months. Note: Assumed to be coverage of “Complementary feeding – education only”.
Immediate initiation of breastfeeding	0%		Not available; set to 0% at baseline.
Lipid-based nutrient supplements	0%		Not available; set to 0% at baseline.
Oral rehydration solution + Zinc	6.6%	[Between countries: median=0.2%; IQR=0-8.1%; range=0-50.7%] LC average=12.3%, LMC average=8.6%, UMC average=1%	LiST^a^ [34].
Public provision of complementary foods	30%	[Between countries: median=25.3%; IQR=0-49.4%; range=0-89.6%] LC average=22%, LMC average=37.5%, UMC average=28.4%	LiST^a^ [34]. Note: Assumed to be coverage of “Complementary feeding – education and supplementation”.
Treatment of SAM	4.7%	[Between countries: median=0%; IQR=0-1.9%; range=0-98%] LC average=12.2%, LMC average=4.4%, UMC average=0%	LiST [34]. Assuming treatment only for children with weight-for-height more than three standard deviations below the WHO Child Growth Standards median (i.e. no management of moderate acute malnutrition). Note: LiST states “Coverage data for this indicator are not typically available. Currently set at 0 for baseline; user should enter local data if possible and available.” Where available in the tool, values for some countries have been used.
Vitamin A supplementation	43.9%	[Between countries: median=48.8%; IQR=9-67.5%; range=0-99%] LC average=71.2%, LMC average=51.7%, UMC average=18.7%	DHS [37]/LiST [34]. Note: Default data used by LiST is from UNICEF [70].
Balanced energy-protein supplementation	0%		Not available; set to 0% at baseline.
Calcium supplementation	0%		Not available; set to 0% at baseline.
Iron and folic acid supplementation for pregnant women	17.2%	[Between countries: median=4.5%; IQR=0-32.9%; range=0-81.5%] LC average=20.6%, LMC average=22%, UMC average=10.7%	LiST^a^ [34]. Note**:** Assumed to be coverage of iron supplementation.
Iron and folic acid supplementation for women of reproductive age	0%		Not available; set to 0% at baseline.
Intermittent preventative treatment of malaria during pregnancy	22.8%	[Between countries: median=17.7%; IQR=0-36.9%; range=0-78.8%] LC average=30.8%, LMC average=19.1%, UMC average=5.1%	LiST^a^ [34]; only includes countries with malaria risk.
Multiple micronutrient supplementation	0%		Not available; set to 0% at baseline.
Iron and folic acid fortification (wheat, maize or rice)	50%		Global estimate from Shekar et al. investment framework for nutrition (2017) [24] Note: Authors state “Baseline coverage of fortification among staple foods (wheat, maize and rice) is based on the existence of legislation status for foods fortified in respective countries. We assume 0 percent if fortification legislation is in the planning stages, 25 percent for voluntary status, and 50 percent if mandatory fortification is legislated.”, citing [71, 72]
Iron and iodine fortification of salt	86%		Global estimate from UNICEF State of the World's Children (2017) [73]
Long-lasting insecticide-treated bed nets	47%	[Between countries: median=54.9%; IQR=16.2-73.9%; range=0-96.7%] LC average=62%, LMC average=39.6%, UMC average=15%	DHS [37]/LiST^a^ [34]; only includes countries with malaria risk.

^a^LiST states “Coverage data for this indicator are drawn from DHS, MICS, and other nationally representative household surveys.”

**Table 3:** Intervention unit costs

Unit costs			
Intervention	*Global average*	*[Between countries: median; IQR and range between countries]* *Low income country (LC), lower-middle income country (LMC) and upper-middle income country (UMC) averages*	Sources and notes for calculating commodity and human resource cost components. Commodity costs have been marked up to include supply chain costs^a^. All costs have been inflated to 2017 US$.
Cash transfers (unconditional)	US$351.41	[Between countries: median=US$286.06; IQR=US$103.97-521.91; range=US$23.74-1182.46] LC average=US$63.22, LMC average=US$230.15, UMC average=US$653.16	Cost per child per annum. Estimated as 10% of per capita GDP.
Delayed umbilical cord clamping	US$2.03	[Between countries: median=US$1.1; IQR=US$0.38-2.79; range=US$0.03-12.85] LC average=US$0.4, LMC average=US$1.4, UMC average=US$3.6	Cost per birth. Assumes 5 minutes of specific health provider time per case^b^ and nurses/midwives receive training every 5 years^c^.
Infant and young child feeding education	US$8.12	[Between countries: median=US$6.63; IQR=US$2.49-12; range=US$0.66-27.03] LC average=US$1.6, LMC average=US$5.4, UMC average=US$15	Cost per child per annum. Country-specific estimates calculated by scaling the cost interval from Shekar et al. investment framework for nutrition [24] according to the range of per capita GDP for the 129 countries. i.e., the lowest cost estimate from Shekar et al. is assumed to be for the country with the lowest GDP per capita, the highest cost estimate for the country with the highest GDP per capita, and cost estimates for each country in between are scaled according to where their GDP per capita falls in the range between the lowest and highest values.
Immediate initiation of breastfeeding	US$21.71	[Between countries: median=US$11.88; IQR=US$3.29-28.26; range=US$0.31-143.79] LC average=US$3.9, LMC average=US$14.5, UMC average=US$40.1	Cost per preterm birth. Assumes 60 minutes of specific health provider time per preterm birth + and nurses/midwives receive training every 5 years^c^.
Lipid-based nutrition supplements	US$23.71	[Between countries: median=US$23.28; IQR=US$22.58-24.22; range=US$21.46-29.75] LC average=US$25.3, LMC average=US$23.1, UMC average=US$23.3	Cost per annum. Commodity costs (US$10, assuming 1/3 sachets/day for 100 days at US$45 for 150 SQ-LNS sachets of 92g^9^) + 18 minutes of specific health provider time per annum (assumed to be the same as for micronutrient powders) [74].
Oral rehydration solution + Zinc	US$2.06	[Between countries: median=US$2; IQR=US$1.91-2.16; range=US$1.72-2.69] LC average=US$2, LMC average=US$1.9, UMC average=US$2.2	Cost per diarrhoea episode. Commodity costs (US$0.77 [74]) + 10 minutes of specific health provider time per case of diarrhoea [74].
Public provision of complementary foods	US$104.48	[Between countries: median=US$94.96; IQR=US$68.46-129.3; range=US$56.78-225.46] LC average=US$62.5, LMC average=US$86.8, UMC average=US$148.4	Cost per child per annum. Country-specific estimates calculated by scaling the cost interval from Shekar et al. investment framework for nutrition (2017) [24] as for IYCF education.
Treatment of SAM	US$246.99	[Between countries: median=US$221.47; IQR=US$187.37-288.85; range=US$86.91-972.69] LC average=US$185.8, LMC average=US$246.3, UMC average=US$288	Cost per case. Commodity costs (US$44.60 for material costs on average^d^, and assuming complicated cases require an average of 14 days, inpatient care was costed according to regional estimates from WHO-CHOICE unit costs of patient services [75]) + 200 minutes of specific health provider time per case of SAM on average^d^. All treatment assumptions are based upon Bhutta et al. (2013)^12^.
Vitamin A supplementation	US$1.36	[Between countries: median=US$1.13; IQR=US$0.57-1.89; range=US$0.33-4.01] LC average=US$0.5, LMC average=US$1, UMC average=US$2.3	Cost per child per annum. Commodity costs (US$0.10 [74]) + 18 minutes of specific health provider time per annum from the OneHealth tool [74].
Balanced energy-protein supplementation	US$54.01	[Between countries: median=US$49.84; IQR=US$38.23-64.89; range=US$33.11-107.02] LC average=US$35.6, LMC average=US$46.3, UMC average=US$73.3	Cost per pregnancy. Country-specific estimates calculated by scaling the cost range from Shekar et al. investment framework for nutrition [24] as for IYCF education intervention above.
Calcium supplementation	US$42.51	[Between countries: median=US$40.65; IQR=US$39.94-44.62; range=US$39.44-54.75] LC average=US$46.8, LMC average=US$41.9, UMC average=US$40.3	Cost per pregnancy. Commodity costs (US$18.60, assuming 1.5g/day for 6 months^15^ at US$0.02/0.3g tablet [76]) + 8 minutes total health provider time per pregnancy [74].
Iron and folic acid supplementation for pregnant women	US$13.78	[Between countries: median=US$13.56; IQR=US$13.07-14.17; range=US$12.57-17.44] LC average=US$14.8, LMC average=US$13.5, UMC average=US$13.4	Cost per pregnancy. Commodity costs (US$5.88 [76]) for 1 tablet per day for 6 months + 8 minutes of specific health provider time per pregnancy [74]. Assumes supplied through community health facilities.
Iron and folic acid supplementation for women of reproductive age	US$1.45	[Between countries: median=US$1.31; IQR=US$1.16-1.63; range=US$1.02-2.58] LC average=US$1.1, LMC average=US$1.3, UMC average=US$1.8	Cost per woman per annum. Commodity costs (US$0.42 [76]) for 1 tablet per week for 3 months + 8 minutes of specific health provider time per pregnancy [74]. Assumes supplied through community health facilities.
Intermittent preventative treatment of malaria during pregnancy	US$0.66	[Between countries: median=US$0.6; IQR=US$0.29-0.96; range=US$0.18-1.54] LC average=US$0.2, LMC average=US$0.4, UMC average=US$1	Cost per pregnancy. Commodity costs (US$0.06 [74]) + 8 minutes specific health provider time per pregnancy [74]. Intervention only applies to countries with malaria risk.
Multiple micronutrient supplementation	US$19.72	[Between countries: median=US$19.34; IQR=US$18.64-20.45; range=US18.11-25.16] LC average=US$21.46, LMC average=US$19.33, UMC average=US$18.94	Cost per pregnancy. WHO regional commodity costs (US$5.52-7.21 [10]) + 8 minutes of specific health provider time per pregnancy [74]
Iron and folic acid fortification (wheat, maize or rice)	US$0.37		Cost per person per annum. Global estimate for wheat flour from Shekar et al. investment framework for nutrition [24] and Horton (2006) [77].
Iron and iodine fortification of salt	US$0.14		Cost per annum. Global estimate from Bhutta et al (2013) [10].
Long-lasting insecticide-treated bed nets	US$4.57	[Between countries: median=US$4.51; IQR=US$4.28-4.73; range=US$4-5.71] LC average=US$4.9, LMC average=US$4.5, UMC average=US$4.4	Cost per person per annum. Commodity costs (US$5.26/3 years [76]) + 5 minutes personnel time for delivery [10]. Long-lasting insecticide-treated bed nets are assumed to last 3 years, so the purchasing cost is $13.05 [$12.37, IQR: $12.10-13.82, range: $11.82-16.88] Intervention only applies to countries with malaria risk.

*Abbreviations*: *DHS* Demographic and Health Survey, *GDP* gross domestic product, *IQR* inter-quartile range, *IYCF* infant and young child feeding, *LC* low income country, *LiST* Lives Saved Tool, *LMC* lower-middle income country, *MAM* moderate acute malnutrition, *OR* odds ratio, *ORS* oral rehydration solution, *RRR* relative risk ratio, *SGA* small for gestational age, *SAM* severe acute malnutrition *SQ-LNS* small quantity lipid nutrient supplement paste, *UMC* upper-middle income country, *WHO-CHOICE* World Health Organization CHOosing Interventions that are Cost-Effective

^a^Country-specific supply chain costs were estimated similarly to Stenberg and colleagues’ [78], who grouped 73 countries into five categories based on “Logistics System Condition”, and estimated a mark-up percentage to apply to commodities for countries in each group. Additional countries in this study were allocated into the five groups by determining an approximate range of GDP per capita for each group (higher GDP per capita is assumed to be associated with better logistics system conditions)

^b^ Hourly (and per minute) wages for staff time estimated for each country by taking per capita GDP, and dividing by an assumed 48 weeks worked per year, and 38 hours worked per week

^c^Training was assumed to cost US$300 per session, with a session educating 10 nurses/midwives every five years. The annual cost per nurse/midwife (US$6) was translated to a per-birth cost by estimating the number of births per nurse/midwife per year: the total number of births^1^ divided by the estimated number of nurses/midwives in the country^2^

^d^All patients are assumed to receive amoxicillin for 5 days (1.5 x 250mg/day at US$0.02/250mg [76]); 15% of cases are assumed to be complicated, requiring inpatient care and receiving 7 days of F-75 therapeutic milk (700mL/day with approximately 2.5L reconstituted milk per 400g carton at US$61.20 per case of 24 cartons [79]). Furthermore, half of complicated cases are assumed to require an additional 14 days of inpatient care and F-100 therapeutic milk (1.4L/day with approximately 2.1L reconstituted milk per 400g carton at US$70.50 per case of 24 cartons [79]). All uncomplicated cases and half of complicated cases also receive 15kg of RUTF over 8 weeks (US$45 for 150 LNS sachets of approximately 100g [75]). For accounting personnel time, uncomplicated cases plus half of complicated cases are assumed to require 10 minutes/week for 8 weeks, and all complicated cases require 60 minutes/day for an average of 14 days

A detailed model description is available in Additional file 1, as well as the Optima Nutrition user guide [80].

### Population and epidemiological data

For 129 countries (selected based on data availability), population and epidemiological data inputs, including baseline data on stunting, wasting and anaemia, were sourced from global datasets and are summarised in Additional file 1, Table B1. The main sources were Demographic and Health Survey (DHS) data [37], Multiple Indicator Cluster Survey (MICS) reports [81], the World Bank Group [32, 33], WHO [82], United Nations Joint Child Malnutrition Estimates (UN-JME) [38], the Global Burden of Disease study [39] and the academic literature [41, 83]. Where country-specific estimates of epidemiological data were not available, estimates (based on regional averages) were taken from the Lives Saved Tool (LiST) [28, 29, 34]

The SDG baseline year (2012) prevalence of stunting and wasting in children were taken from the UN-JME [38] and baseline prevalence of anaemia in women was taken from the Global Burden of Disease study [39]. Annual rates of reduction in undernutrition for each country were obtained from the 2017 Global Burden of Disease SDG indicator projections [39] and applied to baseline (2012) estimates. The Global Burden of Disease study uses statistical methods to project each indicator based on past trends (1990–2017), correlates of these trends with socioeconomic development factors, and expected trends in these socioeconomic development factors [84].

Country-specific inputs are provided in Additional file 2.

### Intervention coverage data

The coverage of interventions (Table 2) was estimated for 2018 for each country from DHS [37] and MICS [81] data. For interventions not directly contained in these surveys, estimates were taken from the LiST [34] model, which creates coverage estimates based on other nationally representative household surveys. For interventions not in DHS, MICS or LiST, baseline coverage was modelled to be zero.

### Intervention cost data

The unit costs of interventions are difficult to estimate because many interventions are integrated within health systems and delivered simultaneously with other health services. Therefore, for this study, we made simple estimates of the country-specific unit costs of each intervention (i.e., the cost per beneficiary) that included costs associated with eight different domains. These were the costs associated with commodities, supply chain and health provider time, as well as fractional costs associated with the additional infrastructure/equipment, health information systems, health financing policy, governance and additional health programme costs that would be required for their expansion.

An ingredient-based approach was used to estimate global commodity costs (estimated from either the WHO International Drug Price Indicator Guide [68] or UNICEF supply cost estimates [71]) and country-specific health provider time requirements (taken from the OneHealth tool [66] and Bhutta et al. (2013) supplement [10], with per capita gross domestic product (GDP) for each country used as a country-specific proxy for salary).

Country-specific supply chain costs were estimated similarly to Stenberg and colleagues’ [70], who grouped 73 countries into five categories based on “Logistics System Condition”, and estimated a mark-up percentage to apply to commodities for countries in each group (ranging from 8 to 50% for drugs and other commodities and 14–63% specifically for insecticide-treated bednets). The additional countries in this study were allocated into the five groups by determining an approximate range of GDP per capita for each group (higher GDP per capita is assumed to be associated with better logistics system conditions).

The fractional costs associated with the remaining five domains were estimated based on Stenberg and colleagues’ [85] work estimating the financing needs of health system expansion to achieve universal health care. They estimated that costs associated with infrastructure and equipment, health information systems, health financing policy, governance and additional health programme costs would comprise 51% of the total costs (i.e. commodities, health provider time and supply chain costs only account for 49% of all costs). Therefore, the overall (country-specific) unit cost for each intervention was calculated by inflating the commodity, health provider time and supply chain costs by ~ 100%.

Costs are presented in 2017 US$ with details for each intervention in Table 3. Discounting is not included.

### Status quo scenario: maintained existing investment

The model was run without changes to intervention coverage, thus including only continued current trends in stunting and wasting in children and anaemia in women (based on annual rates of reduction for each country, obtained from the 2017 Global Burden of Disease SDG indicator projections [78] and applied to baseline (2012) estimates). The models were projected from 2019 to 2030, and progress was measured against the SDG targets.

### Maximum impact scenario

For each country, a projection was run where a 5-year period was used to linearly scale up all interventions from their baseline estimated coverage in 2019 to 95% coverage of their target population in 2024, which was maintained in the model until 2030. The effect sizes of interventions were assumed to be the same across countries as insufficient evidence is available on how they vary by setting.

### Intervention expansion pathways

For each country, scenarios were run with each of the 18 interventions scaled up one at a time to 95% coverage over a 5-year period (2019–2024) and maintained until 2030. In each scenario, the total additional cost and impact on each indicator (stunting, wasting, anaemia) was recorded and compared to the status quo, with the average cost-effectiveness of each intervention calculated as the additional cost divided by the number of cases averted over the period 2019–2030 (e.g. average cost per stunting case averted). For each country and indicator, the single most cost-effective intervention was identified.

To identify the second most cost-effective intervention, the results above are inadequate as the effects of scaling interventions together will not be additive. This is because intervention effectiveness measures are based on *relative reductions* in nutritional outcomes, and so to avoid overestimating combined impact, the second intervention is modelled against a new baseline where the first intervention has already reduced a risk factor or outcome. This is consistent with other nutrition models [29, 86]. Therefore, to identify the next most cost-effective intervention for each indicator, the process above was repeated but with each of the remaining 17 interventions scaled up one at a time to 95% coverage, alongside the most cost-effective intervention, with outcomes compared to the scenario of only the most cost-effective intervention being scaled up.

This methodology was repeated to identify a sequence of interventions for each indicator that represent a prioritisation for inclusion in an overall package of interventions.

### Uncertainty bounds

Univariate uncertainty analyses were conducted to generate uncertainty intervals for the estimated impact (obtained by running projections using the lower and upper bounds of their effect size estimates) and costs (lower bounds based on commodity costs of interventions only, and upper bounds assuming double the non-commodity costs) of all interventions and countries.

### Sensitivity analyses

Univariate sensitivity analyses were used to explore a variety of alternate analyses including the following: the possible impact if only nutrition-sensitive interventions were considered (i.e. excluding cash transfers, intermittent preventative treatment of malaria during pregnancy (IPTp), long-lasting insecticide-treated bed nets (LLINs)), comparison against the extended 2030 global targets [87] (50% reduction in the number of stunted children, wasting in children < 3%, 50% reduction on anaemia in women); and sub-analyses for low-income, lower-middle-income and upper-middle-income countries.

## Results

**Fig. 1:**
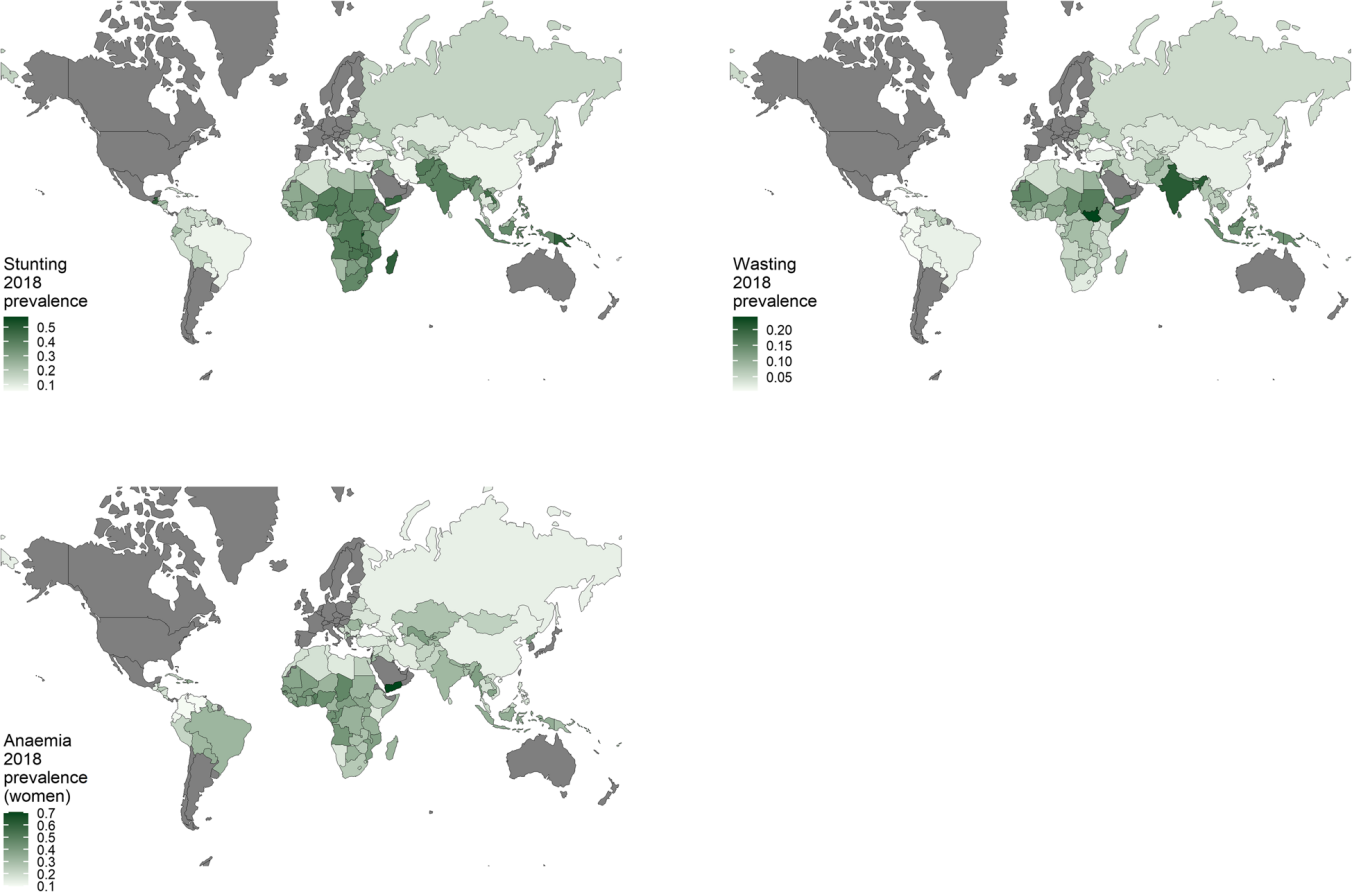
Estimated 2018 prevalence of stunting in children under 5 (top left), wasting in children under 5 (top right) and anaemia in women of reproductive age (bottom left)

The estimated 2018 prevalence of undernutrition derived for the 129 countries varied significantly by country and indicator, but was generally highest in south and central Africa (Fig. 1). Under-five stunting prevalence was highest in Burundi at 57% (global average 25%), under-five wasting prevalence was highest in South Sudan at 24% (global average 6%) and anaemia prevalence among women was highest in Yemen at 71% (global average 29%).

Under the status quo (i.e. no change to current trends), 46 (34%) of the 129 countries were on track to achieve a 40% in reduction in stunting by 2025, 66 (51%) were on track to reduce wasting to below 5% by 2025 and 0 (0%) were on track to reduce anaemia by 50% by 2025.

**Fig. 2:**
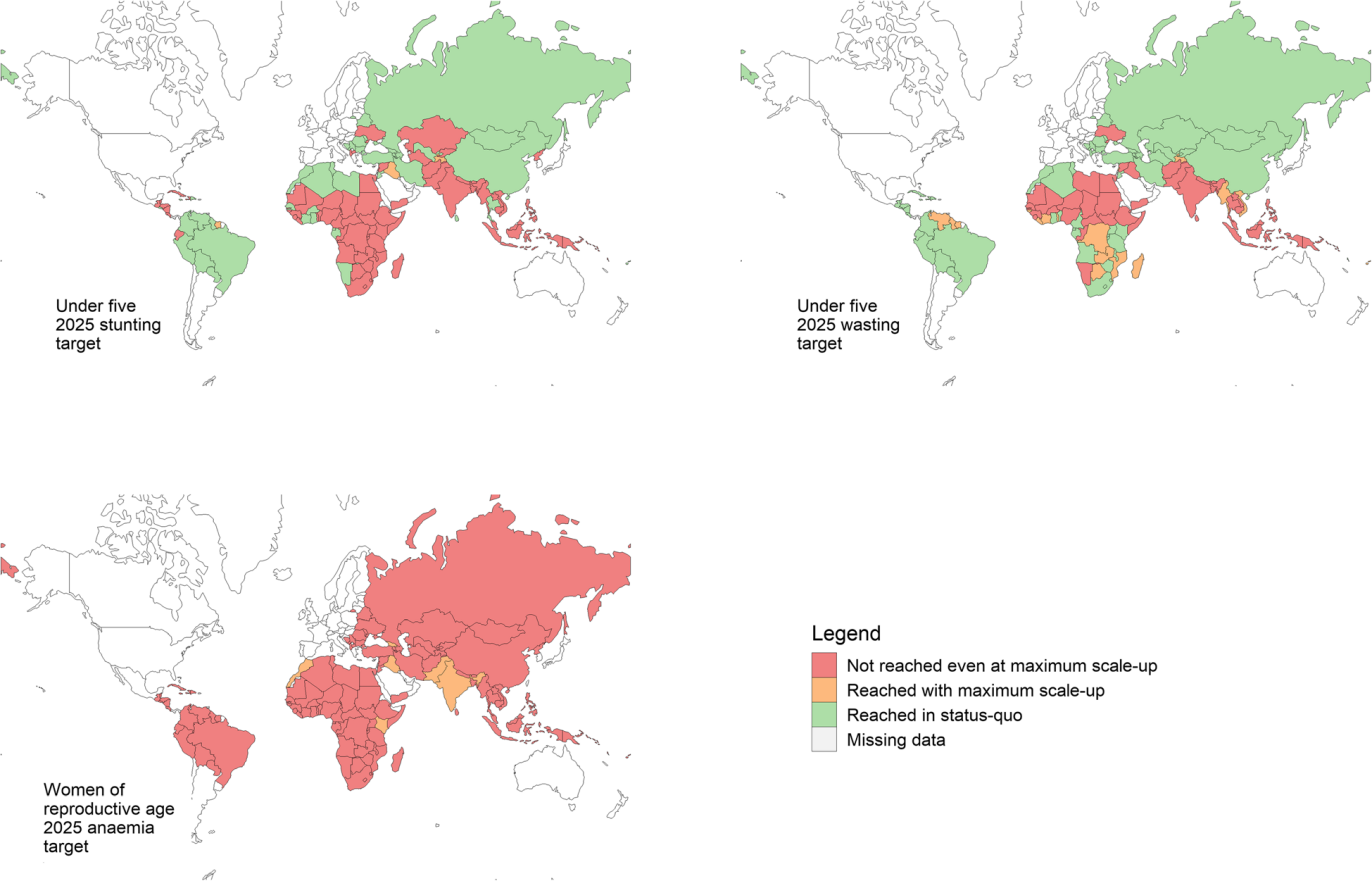
Countries that are projected to reach targets under that status quo (green), in the maximum impact scenario (orange) or not at all (red). Panels show targets for stunting in children under five (top left), wasting in children under five (to-right) and anaemia in women of reproductive age (bottom left)

**Fig. 3:**
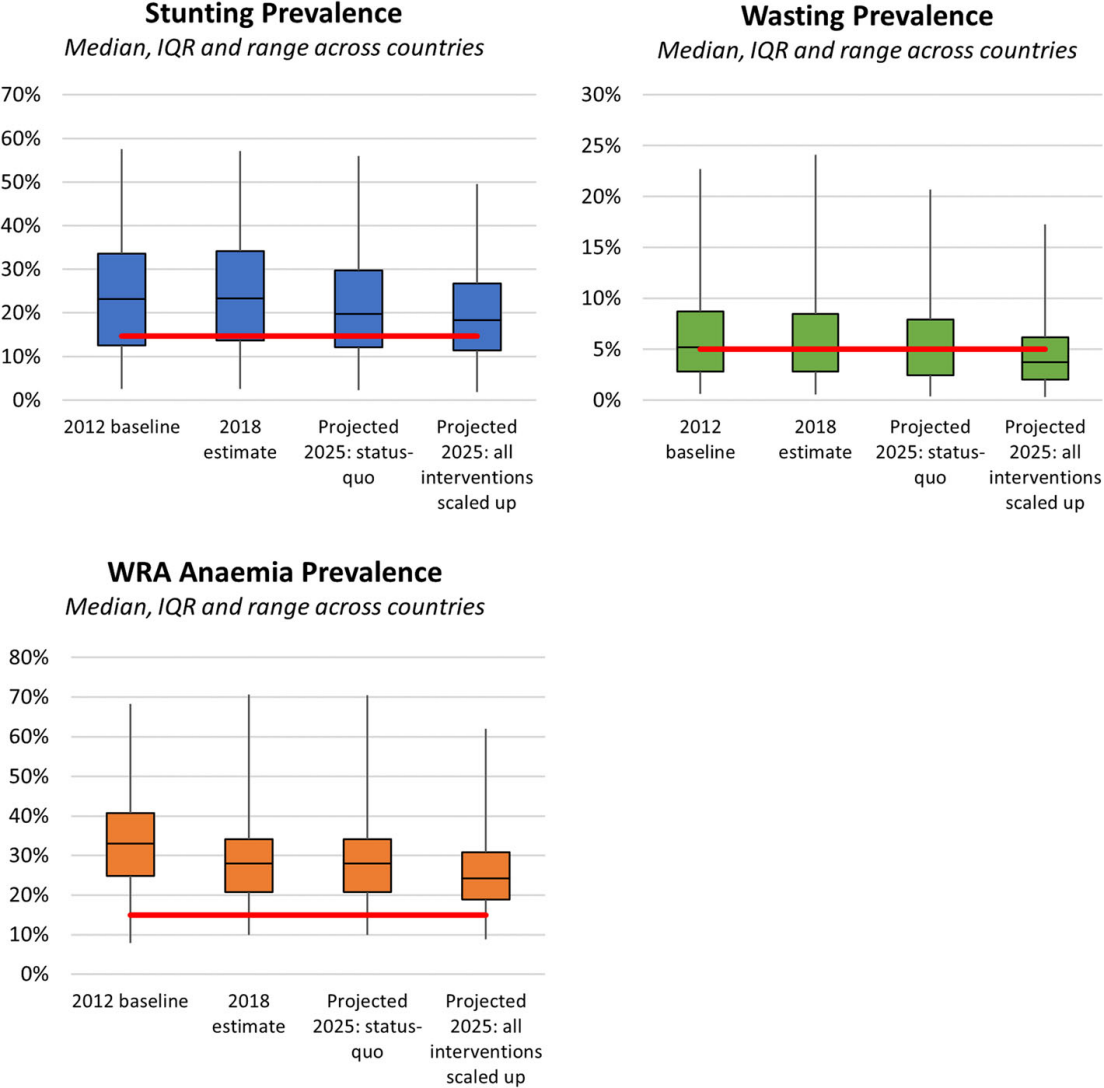
Projected changes to nutrition indicators under the status quo and maximal impact scenarios for individual countries. Boxplots show the median and inter-quartile range of indicators across countries, with tails representing the maximum and minimum values. The red line represents the relevant 2025 target at a *global* level. **a** Stunting prevalence among children under five. **b** Wasting prevalence among children under five. **c** Anaemia prevalence among women of reproductive age. Abbreviations: IQR, inter-quartile range; WRA, women of reproductive age

Scaling up all 18 nutrition interventions to 95% coverage reduced the cumulative number of children reaching age five stunted or wasted between 2019 and 2030 by 42.1 million and 13.8 million respectively, and averted 476 million cases of anaemia. With all interventions scaled up, the global number of children under five who were stunted in 2030 was 20% lower than the status quo scenario in 2030, and the prevalence of wasting in children and anaemia in women in 2030 were reduced by an average of 14% and 22%, respectively, compared to 2030 prevalence in the status quo scenario. With all interventions scaled up, an additional 4, 17 and 7 countries were projected to reach the stunting, wasting and anaemia targets respectively (Fig. 2, with projected stunting prevalence in Fig. 3).

**Fig. 4:**
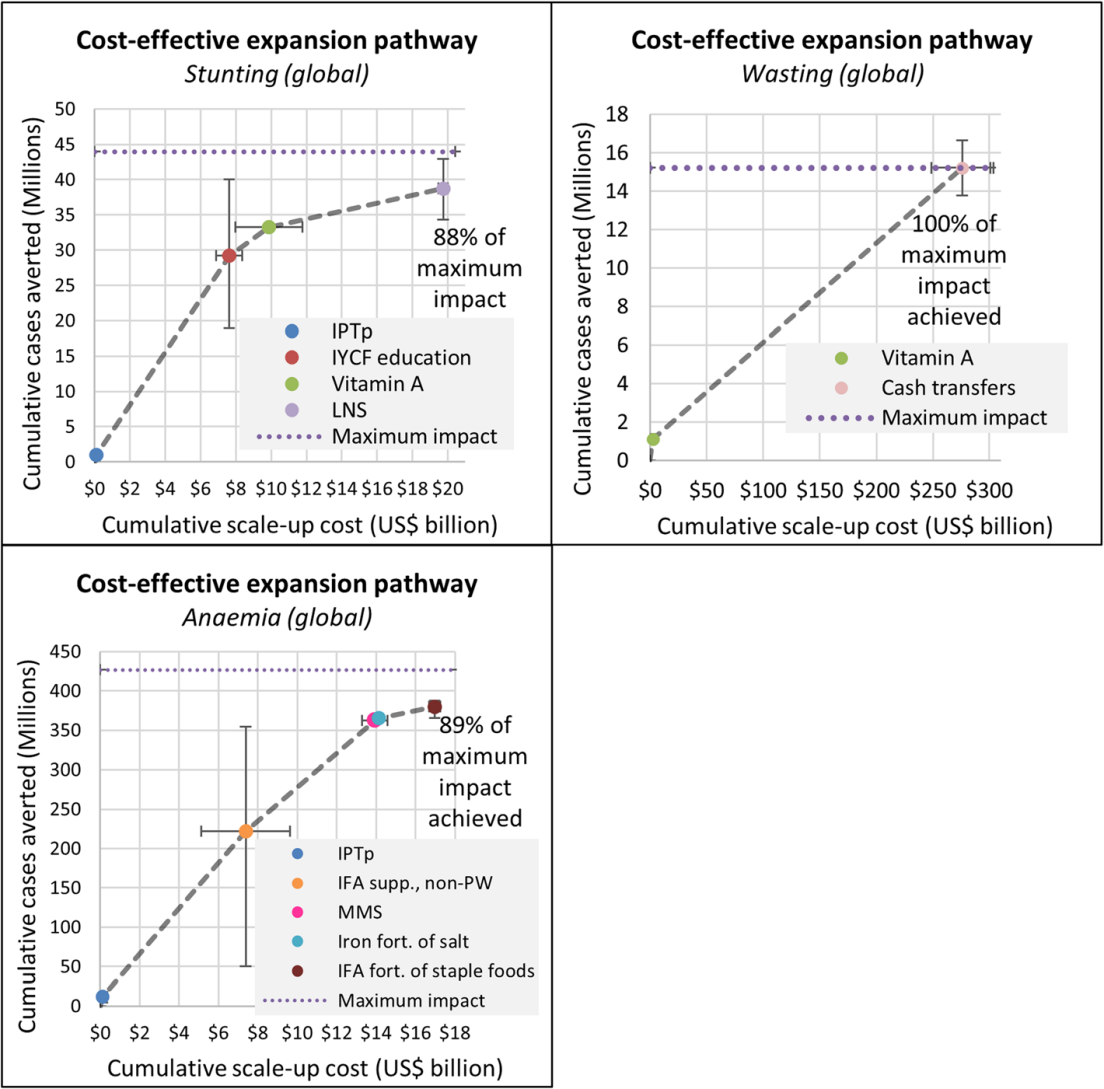
Cost-effective expansion pathway for reducing the prevalence of stunting in children under five (top left), wasting in children under five (top right) and anaemia in women of reproductive age (bottom left). The additional cost and impact at sequential steps are calculated by taking continued status quo outcomes 2019–2030 aggregated over all countries, and sequentially increasing intervention coverage in all countries from baseline to 95% over a 5-year period (2019–2024). Variations in prioritisation exist for individual countries (Additional file 2). Maximum impact is the total cases averted across all countries when all 18 interventions were scaled up simultaneously. Abbreviations: IFA, iron and folic acid; IFA supp., iron and folic acid supplementation; IPTp, intermittent preventative treatment of malaria during pregnancy; IYCF, infant and young child feeding; LNS, lipid-based nutrition supplements; MMS, multiple micronutrient supplementation; PW, pregnant women

Scaling up all interventions was estimated to cost an additional $458 billion between 2019 and 2030; however, the majority of the total possible impact came from only a few interventions (Fig. 4). At an aggregate global level, the model estimated that scaling up IPTp (in regions with malaria), infant and young child feeding (IYCF) education, vitamin A supplementation and lipid-based nutrition supplements produced 88% of the total global impact on stunting and cost US$19.75 billion between 2019 and 2030, with average costs per case averted of US$103, US$267, US$556 and US$1795 when interventions were consecutively added, respectively (Table 4). In a sensitivity analysis, we estimate that if IYCF were more than 2.1 times our unit cost estimate, then the order of IYCF and vitamin A supplementation would be reversed. Country-specific estimates are provided in Additional file 2.

**Table 4:** Cost-effective expansion pathways for stunting, wasting and anaemia in prevalence in children under five. Values in parentheses represent uncertainty bounds

		Additional cases averted^a^ when intervention was added, 2019-2030 (million)	Percentage of total impact (i.e. impact with all interventions)	Additional cost to expand to 95% coverage 2019-2030 (billion US$)	Average cost per impact (cost per case averted when intervention was added to package)
Order	Stunting intervention				
1	IPTp	1.00 (0.64 - 1.31)	2.3%	US$0.10 (0.04 - 0.17)	**US$103**
2	IYCF education	28.22 (17.95 - 39.04)	64.2%	US$7.52 (6.77 - 8.27)	**US$267**
3	Vitamin A supplementation	4.04 (3.61 - 4.48)	9.2%	US$2.24 (0.35 - 4.14)	**US$556**
4	Lipid-based nutrient supplements	5.50 (1.06 - 9.71)	12.5%	US$9.88 (9.61 - 10.15)	**US$1,795**
	*Cumulative*	38.76 (23.25 - 54.53)	88.1%	US$19.75 (16.77 - 22.72)	
Order	Wasting intervention				
1	Vitamin A supplementation	1.13 (0.98 - 1.29)	7.4%	US$2.24 (0.35 - 4.14)	**US$1,989**
2	Cash transfers	14.09 (12.66 - 15.52)	92.6%	US$273.73 (246.35 - 301.10)	**US$19,427**
	*Cumulative*	15.22 (13.64 - 16.81)	100.0%	US$275.97 (246.71 - 305.23)	
Order	Anaemia intervention				
1	IPTp	11.42 (4.62 - 17.76)	2.7%	US$0.10 (0.04 - 0.17)	**US$9**
2	IFA supplementation for non-pregnant women	210.51 (38.98 - 343.05)	49.3%	US$7.28 (5.03 - 9.53)	**US$35**
3	Multiple micronutrient supplementation	140.43 (62.93 - 177.88)	32.9%	US$6.55 (5.90 - 7.21)	**US$47**
4	Iron fortification of salt	2.97 (0.00 - 4.54)	0.7%	US$0.21 (0.19 - 0.23)	**US$71**
5	IFA fortification of staple foods	14.61 (0.00 - 22.20)	3.4%	US$2.83 (2.55 - 3.12)	**US$194**
	*Cumulative*	379.94 (106.54 - 565.44)	89.0%	US$16.98 (13.70 - 20.25)	

^a^Measured as difference in cumulative number of children turning age five stunted or wasted between 2019-2030, and difference in the sum of anaemic women per year between 2019-2030

*Abbreviations*: *IFA* iron and folic acid, *IPTp* Intermittent preventative treatment of malaria during pregnancy, *IYCF* infant and young child feeding

At an aggregate global level, scaling up vitamin A supplementation and cash transfers produced 100% of the total impact on wasting and cost US$275.97 billion between 2019 and 2030 (predominantly for cash transfers), with average costs per case averted of US$1989 and US$19,427 when interventions were consecutively added, respectively (Table 4). Note that while the treatment of severe acute malnutrition (SAM) intervention is effective at preventing mortality from wasting, in the model it had no impact on wasting prevalence (Table 1 and “Discussion”).

At an aggregate global level, scaling up IPTp, iron and folic acid (IFA) supplementation for non-pregnant women, multiple micronutrient supplementation for pregnant women produced 89% of the total impact on anaemia and cost US$16.98 billion between 2019 and 2030, with average costs per case averted of US$9, US$35, US$47, US$71 and US$194 when interventions were consecutively added, respectively (Table 3). Iron fortification of salt and iron and folic acid fortification of staple foods were the next most cost-effective for reducing anaemia.

There were important differences between countries as to which interventions were the most cost-effective, which were driven largely by differences in the data inputs such as risk of malaria (countries without malaria receive no benefit from IPTp or LLINs), prevalence of breastfeeding (higher breastfeeding prevalence reducing the impact of IYCF education), incidence of diarrhoea (less diarrhoea meaning reduced impact of vitamin A supplementation), the proportion of small and preterm births (smaller impact of supplements for pregnant women) and unit cost assumptions. However, despite differences in the prioritisation of interventions between settings, the finding that the majority of the total impact came from a select few interventions remained true.

Sub-analyses indicate that the majority of the cost and impact is in low-income countries, rather than lower-middle- or upper-middle-income countries (Table 5, based on World Bank classifications). Similar results were found when progress was assessed against the 2030 targets. If only nutrition-specific interventions were included in the analysis (i.e. excluding cash transfers, IPTp, LLINs), then a smaller impact was achieved, particularly for wasting and anaemia, highlighting the benefits of these interventions.

**Table 5:** Model sub-analysis projections for the number of countries reaching the SDG 2.2 targets. Each row represents the maximum impact scenario, where interventions are scaled up to 95% coverage in each country over a five-year period. The main analysis row is the same as the results presented above, and other rows are sensitivity analyses

	# countries reaching target			# cases averted in max impact scenario			Total additional cost 2019-2030 (billion)
	Stunting	Wasting	Anaemia	Stunting	Wasting	Anaemia	
Main analysis	50/129	83/129	7/129	42,106,000	13,783,000	476,304,000	US$458
No nutrition-sensitive interventions (i.e. excluding cash transfers, IPTp, LLINs)	49/129	70/129	4/129	41,323,000	806,000	436,170,000	US$155
2030 targets instead of 2025 targets	8/129	53/129	7/129	42,106,000	13,783,000	476,304,000	US$458
Low income countries only	9/33	18/33	2/33	12,172,000	3,641,000	94,488,000	US$58
Lower-middle income countries only	15/46	25/46	1/46	24,917,000	9,550,000	274,880,000	US$244
Upper-middle income countries only	26/50	40/50	4/50	5,017,000	592,000	106,936,000	US$156
Interventions scaled up over 10 years instead of 5 years	44/129	74/129	7/129	29,007,000	10,122,00	355,187,000	US$337

*Abbreviations*: *IPTp* Intermittent preventative treatment of malaria during pregnancy, *LLINs* long-lasting insecticide-treated bednets, *SDG* Sustainable Development Goal

## Discussion

Using the Optima Nutrition model, scaling up 18 evidence-based nutrition interventions to 95% coverage across 129 countries could lead to 42 million fewer stunted children between 2019 and 2030 and a 20% reduction in the number of children stunted in 2030, and reduce the 2030 prevalence of wasting in children and anaemia in women by an average of 14% and 22% respectively. This work expands previous analyses by identifying a subset of interventions that are the most cost-effective and contribute the greatest impact towards the SDG undernutrition indicators; these interventions should be prioritised in the context of limited financial resources. These models also highlight the need to consider a broader set of interventions to address food systems and social determinants of health in order to reach the SDG targets, for example agriculture interventions and interventions for poverty alleviation, food security, WASH and women’s empowerment. This is consistent with previous studies that have recognised the that nutrition-specific interventions alone will not help countries reach SDG targets, highlighting the need for investments in evidence-based nutrition-sensitive interventions [5, 10, 18, 23, 24, 88].

For reducing stunting, IPTp, IYCF education and vitamin A supplementation were the most cost-effective interventions globally. IPTp can lead to improved birth outcomes in areas with malaria risk [66], therefore reducing stunting risk, but it was its low cost rather than high impact that made it the most cost-effective intervention—even at high coverage it produced limited total gains. IYCF education can improve breastfeeding and complementary feeding behaviours [45], which can reduce diarrhoea [45] and stunting [89]. Importantly, relevant and high-quality education must be delivered that includes support for breastfeeding at a local and national level, combined with enabling activities such as countries implementing WHO codes for breast-milk substitutes [90]. The next most cost-effective intervention for stunting was vitamin A supplementation [91]. Vitamin A supplementation has been implemented at scale in many countries already to both reduce mortality and prophylactically reduce diarrhoea incidence, and this study provides evidence to continue expansion of this intervention to countries where this is not already the case.

For reducing wasting, vitamin A supplementation was identified as the most cost-effective intervention. Vitamin A supplementation can reduce diarrhoea incidence, a risk factor for wasting, and is therefore preventative. While the treatment of SAM intervention is effective at preventing mortality from wasting [10, 59], in the model it had no impact on wasting prevalence. This is because the intervention is defined as treating children until they reach a weight-for-height of three standard deviations below the WHO Child Growth Standards median, at which point their mortality risks are significantly reduced but they are still defined as being wasted for purposes of the SDG target (i.e. children who are severely wasted are treated to become only moderately wasted, but wasted nonetheless). Cash transfers were identified as the next most cost-effective intervention, but with a cost per case averted six times higher than vitamin A supplementation. Cash transfers is an example of a social protection intervention primarily intended to help households meet basic needs; however, this study provides evidence of its potential benefit for nutrition. For anaemia, it is not surprising that IPTp, IFAS for non-pregnant women and multiple micronutrient supplements for pregnant women were the most cost-effective given their low unit costs and high impacts (Table 1).

The results of this study are consistent with previous work, but expand the intervention and country sets, and also consider intervention prioritisation. Other studies have estimated that scaling up subsets of these 18 nutrition interventions could lead to approximately a 20% reduction in stunting in children [23, 24] (~ 65 million cases averted globally [24]). The consistency of impact projections is not surprising because the effect estimates for interventions (Table 1), as well as the causal pathways and risk factors used in the Optima Nutrition model (Figure A2), are based on the same evidence base as other models used for these analyses (e.g. LiST [92, 93]). A recent study also estimated that 45% and 35% of 105 LMICs analysed were likely to reach the stunting and wasting targets respectively [94], which is consistent with our analysis if we constrain to the same set of countries. Our cost estimate of approximately $42 billion per annum to scale up all 18 interventions was more than previous estimates ($10.3 billion [23] and $9.6 billion [10] per annum, and $70 billion over 10 years [24]), because, as well as including additional high-cost interventions (e.g. cash transfers), our unit costs are up to twice as high due to the fractional costs associated with infrastructure and health system strengthening. Where comparisons are available, the cost-effectiveness estimates for individual interventions are approximately in line with World Bank estimates (e.g. $266 and $467 per stunting case averted for vitamin A and IYCF education respectively [24], and $10–62 per anaemia case averted with IPTp and micronutrient supplements for women [24]).

Between countries, different intervention priorities may be needed based on differences in baseline nutrition indicators, baseline intervention coverages, intervention effect sizes and intervention costs. In this analysis, effect estimates for interventions and baseline intervention coverage did not have an impact on between-country variation in cost-effectiveness. This is because intervention effect sizes were assumed to apply across all settings, a limitation of this study due to lack of evidence indicating otherwise, and because a linear cost-coverage relationship was used, meaning that baseline intervention coverage affected the impact that could be achieved with further scale-up but not cost-effectiveness. In this analysis, country-specific unit costs were calculated for interventions; however, between countries the intervention costs were largely scaled in proportion to one another (e.g. the human resource component costs were scaled according to the GDP) meaning that the relative costs were generally unchanged. Therefore, the greatest driver of between-country differences was baseline nutrition indicators, highlighting the importance of tailoring interventions to target areas or risk factors that drive the greatest burden (e.g. IYCF education was a higher priority in settings with lower baseline breastfeeding prevalence).

Major areas of current and future research are to define a broader set of interventions that can indirectly improve nutrition indicators, and to quantify their costs and benefits. For example, there is evidence that animal-sourced foods may reduce stunting [95, 96], but there are limited studies linking agriculture and food system interventions to changes in nutritional indicators. Addressing the known underlying determinants of undernutrition is also critical. For example, improving gender equity and ensuring women can choose when and how many children they have can improve financial security and reduce poverty [97, 98], both of which are correlated with poor nutritional outcomes. However, defining interventions to empower women and quantifying their effect sizes, which will be highly context specific, remains a challenge.

### Implications of the COVID-19 pandemic

The COVID-19 pandemic is expected to have profound impacts on countries’ ability to achieve the SDG targets [99]. Recent estimates suggest that globally there could be a 14.3% increase in the prevalence of moderate or severe wasting among children under five in 2020, or 6.7 million additional children with wasting, compared with projections for 2020 without COVID-19 [100]. The implications for stunting are less clear, as it is a chronic condition and would therefore depend on the duration of the pandemic and associated disruptions.

In this study, the subset of interventions identified as the most cost-effective is based on pre-COVID-19 estimates of nutrition indicator trends, baseline intervention coverages and intervention unit costs. In particular, short-term changes to the unit costs (e.g. if commodities are more difficult to procure, if there are additional personal protective equipment costs or if services are delivered differently), if large enough and disproportionate across interventions, may lead to changes in the prioritisation of interventions. Further work is required to identify how to prioritise interventions to address the acute impacts of COVID-19, and this is currently being undertaken by the *Standing Together for Nutrition* consortium [101]. Once the immediate impacts of COVID-19 are managed, costs may revert to pre-pandemic levels, but at this point, we cannot predict when this will occur.

### Limitations

This study has a number of limitations. The effect sizes of interventions may be overestimated as they were taken from meta-analyses that included an overrepresentation of randomised controlled trials. The modelled impact is therefore based on the assumption that interventions could be implemented as effectively observed in controlled conditions, and delivered precisely to their target populations, which is not likely to be the case. Therefore, our lower-bound estimates, which were derived from the lower-bound impact estimates, may be closer to the actual achievable impact when accounting for loss of effectiveness when moving from trials to scaled up programmes. Effect estimates were assumed to apply across all settings, due to lack of evidence indicating otherwise, but in reality, some populations will respond to them differently. We did not assume any complementary effects of interventions when implemented together, and applied consecutive independent relative reductions, but it is unclear whether there are cumulative benefits (or diminishing returns) to layering interventions.

It is unclear how feasible it would be to scale some of these interventions to high coverage, particularly within 5 years. The coverage of interventions was assumed to be constant in the status quo, but may naturally increase as health systems are strengthened, which would mean we have overestimated the impact of scaling up these interventions (but the cost-effectiveness would remain as estimated). Similarly, changes in projected populations sizes, GDP growth, poverty and other indicators may influence the baseline (status quo) projection, and hence the results. Where no intervention coverage data was available, we assumed a baseline zero coverage, which would also mean we have overestimated the impact of scale-up if some coverage already existed.

There are limitations to the model structure. For example, the model is based on the risk factor structure / causal pathway outlined in Figure A2 based on evidence available to support and quantify each relationship. Optima Nutrition is a global model and as such relies on a high threshold of evidence for interventions and/or risk factors to be included (i.e. typically meta-analyses of randomised controlled trials). This means that some interventions, particularly those that impact risk areas not in Figure A2, may be being overlooked where trial data are unpublished or no meta-analysis exists because few published trials are available.

While costing studies exist for selected interventions in selected countries, nothing is currently available at a global level that could be adapted across the 129 settings that were modelled. Therefore, we generated simple unit cost estimates that attempted to include the different costing domains, but individual country-specific costing studies could more precisely account for staffing, infrastructure, logistic and other overhead costs and improve the accuracy of cost and cost-effectiveness estimates. We also assumed that the unit costs of interventions would remain constant with scale, which may not be the case as economies of scale may reduce marginal costs as coverage increases and saturation effects may decrease marginal costs as coverage becomes high.

Population and epidemiological data inputs came largely from global data sets, and for some countries, this required imputing regional values or using modelled data where estimates were missing. This also does not account for differences within each country by geographic location or wealth quintile, or temporal differences such as seasonality that may be associated with wasting but not captured in DHS or MICS surveys as a result of survey timing. In the main results, we have rolled-up country estimates to derive global progress; however, there is a great deal of subnational variation in progress and our supplementary results may be more useful for individual countries than whether or not global targets are reached.

## Conclusions

Of the 129 countries modelled, 46 (34%) were on track to achieve a 40% in reduction in stunting by 2025, 66 (51%) were on track to reduce wasting to below 5% by 2025, and 0 (0%) were on track to reduce anaemia by 50% by 2025. Scaling up 18 nutrition interventions globally could reduce stunting, wasting and anaemia by 20%, 14% and 22% respectively, and increase the number of countries on track to achieve the SDG 2.2 targets to 50 (39%), 83 (64%) and 7 (5%) respectively. The majority of the total impact was the result of only nine interventions, which suggests that they should be prioritised in the context of limited budgets.

## Supplementary information

**Additional file 1.** Detailed model description and additional model parameters.

**Additional file 2.** Country-specific model inputs and country-specific model results.

## Data Availability

Model inputs and parameters are available in the supplementary material.

## Abbreviations

DHS: Demographic and Health Survey
GDP: Gross domestic product
IFA: Iron and folic acid
IYCF: Infant and young child feeding
IQR: Inter-quartile range
IPTp: Intermittent preventative treatment of malaria during pregnancy
LiST: Lives Saved Tool
LLINs: Long-lasting insecticide-treated bed nets
MAM: Moderate acute malnutrition
MICS: Multiple Indicator Cluster Survey
SDG: Sustainable Development Goal
SGA: Small for gestational age
SAM: Severe acute malnutrition
UN-JME: United Nations Joint Child Malnutrition Estimates
WASH: water, sanitation and hygiene
WHO: World Health Organization
