# Supplementary material for: Ending malnutrition in all its forms requires scaling up proven nutrition interventions and much more: a 129-country analysis
Source: BMC Med. 2020 Nov 13;18:356. doi: 10.1186/s12916-020-01786-5 (PMC7661178; doi:10.1186/s12916-020-01786-5)
Supplement: Supplementary file 1 — Additional file 1. Detailed model description and additional model parameters. [file 12916_2020_1786_MOESM1_ESM.pdf]

APPENDIX A. Detailed model description

A.1 Overview of populations and risk factors

The Optima Nutrition model tracks the number of women of reproductive age (15-49 years) in a population, who can become pregnant and give birth. After birth, children are tracked until five years of age across five age bands: <1 month, 1-5 months, 6-11 months, 12-23 months and 24-59 months (Figure A.1).

Children in each age-band are categorised by height-for-age (stunting) status, weight-for-height (wasting) status, anaemia status, breastfeeding practice, and economic status (above or below the poverty line). Women of reproductive age are classified by anaemia status.

Children exit the model either when they reach the age of 60 months or by death, which can happen at any age. Children in the < 1 month age-band can die due to diarrhoea, pneumonia, meningitis, asphyxia, sepsis, prematurity and "other" causes, while children in all other age bands can die of diarrhoea, pneumonia, measles and "other" causes ("other" causes is used to capture, and match to, population statistics of known overall mortality rates for the given application context). The relative risks of dying from each cause are related to the child's breastfeeding, height-for-age, weight for height, and anaemia status. Mortality is also tracked for pregnant women, who can die from antepartum haemorrhage, intrapartum haemorrhage, postpartum haemorrhage, hypertensive disorders, sepsis, abortion, embolism, other direct causes and other indirect causes. The relative risks of pregnant women dying from haemorrhage are related to their anaemia status.

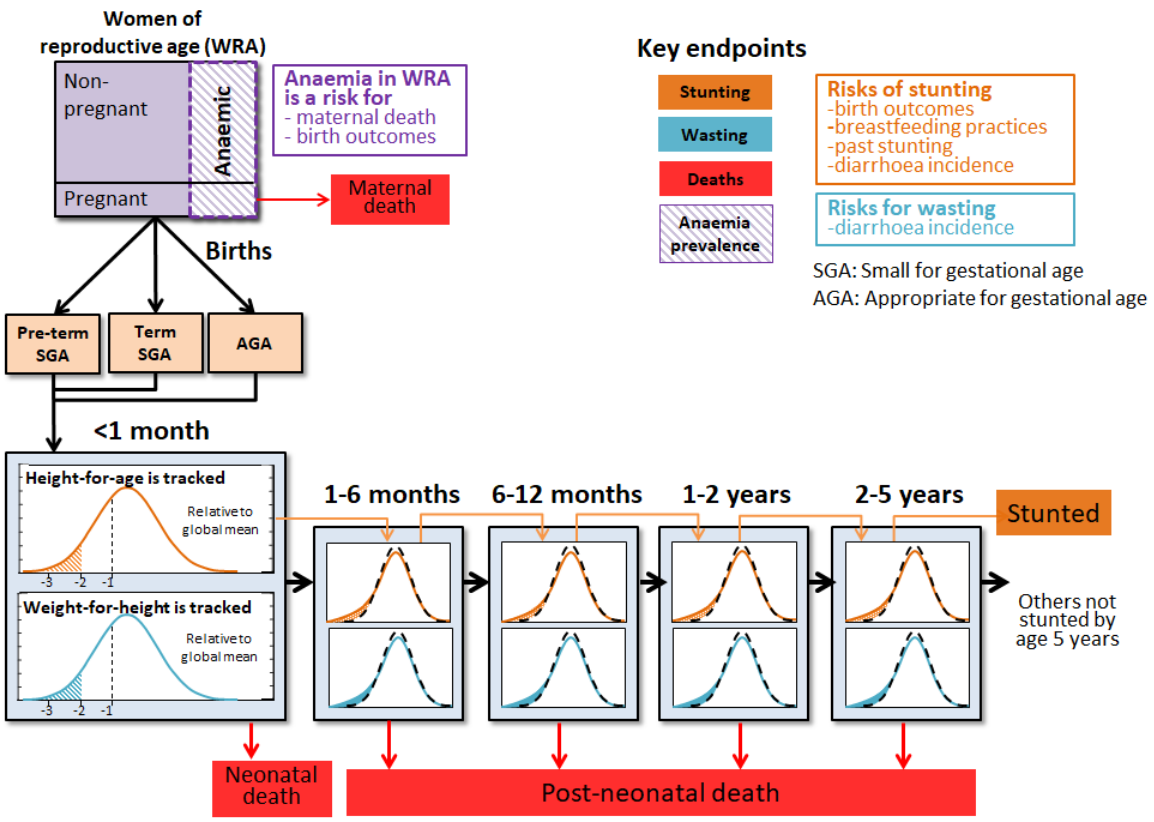

Figure A.1: Populations within the Optima Nutrition model

Several risk factors for stunting in children are modelled: birth outcomes (pre-term birth and/or a child being born small for gestational age [SGA]), stunting in a previous age-band, suboptimal feeding practices (age-appropriate breastfeeding and complementary foods), and incidence of diarrhoea (Figure A.2). In addition, anaemia in women of reproductive age is modelled to be a risk factor for sub-optimal birth outcomes; birth outcomes and diarrhoea incidence are modelled to be risk factors for wasting; and sub-optimal breastfeeding is modelled to be a risk factor for diarrhoea incidence.

In the model, interventions can improve nutritional outcomes directly or indirectly by reducing risk factors. For example, Figure A.2 shows that changes to breastfeeding practices, perhaps through better education, can directly reduce mortality and diarrhoea incidence. Moreover, in the model this will also lead to an indirect reduction in mortality because a reduction in diarrhoea incidence will lead to a reduction in stunting and wasting, which will subsequently further reduce mortality. Changing the coverage of an intervention among its target population leads to changes in projected outcomes based global estimates of intervention effectiveness.

In previous work [27], the epidemiological component of the Optima Nutrition model has been validated by comparing the effects of scaling up the included interventions to those estimated by the Lives Saved Tool (LiST) [28], and found to be in good agreement. This is not surprising as both models use the same underlying impact pathway [29], based on the latest available evidence. The Optima Nutrition model is newer than the LiST model, however the LiST model has been used and validated against real-world outcomes in sub-Saharan Africa, finding it to be a useful tool despite limitations in data availability [30, 31].

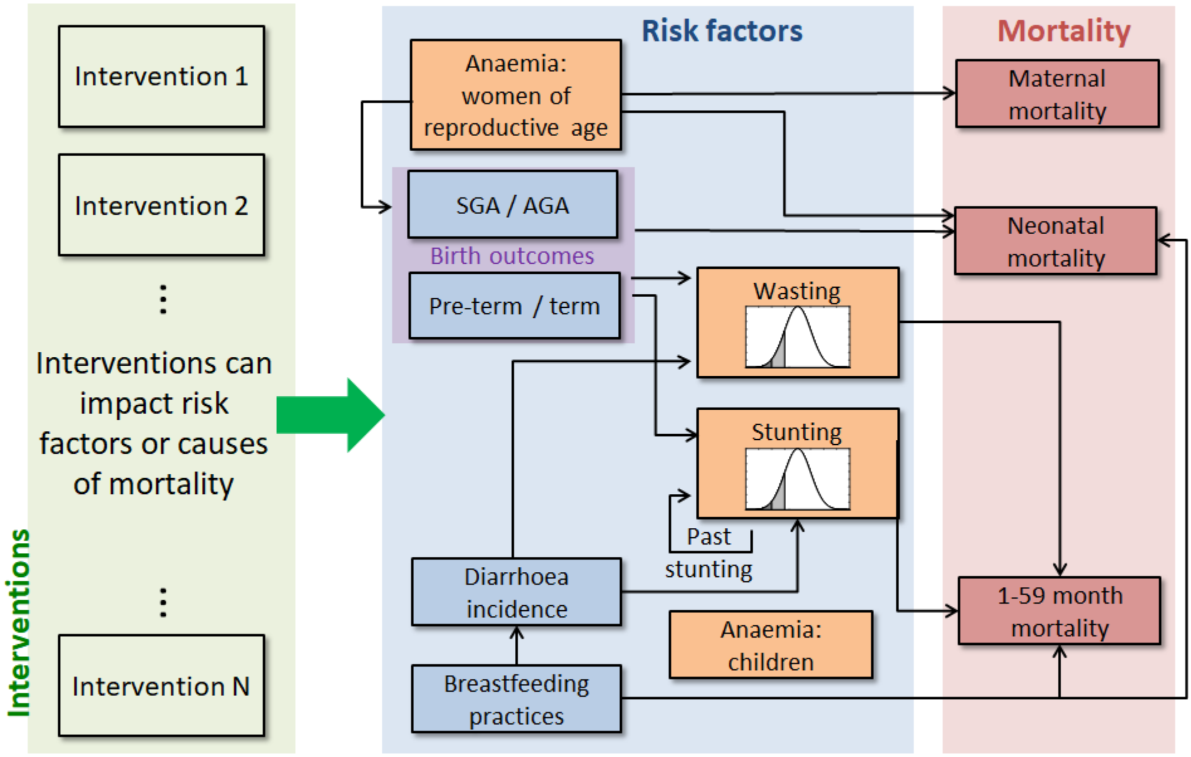

Figure A.2: The relationship between interventions, risk factors and mortality. SGA, small for gestational age; AGA, appropriate for gestational age.

Optima Nutrition uses an economic model to translate the amount spent on an intervention to its estimated coverage. For each intervention, this requires a setting-specific input for the unit cost.

A.2 Modelling stunting using Optima Nutrition

- The model divides children in each age-band into four height-for-age categories, based on WHO criteria (Figure A.**3**), with the two lowest categories (severe and moderate) being considered stunting:
    - Severe: < -3 standard deviations below the median height-for-age of the WHO reference population
    - Moderate: < -2 & >= -3 standard deviations below the median height-for-age of the WHO reference population
    - Mild: < -1 and >= -2 standard deviations below the median height-for-age of the WHO reference population
    - Normal: >= -1 standard deviation below the median height-for-age of the WHO reference population
- Risk factors for stunting are suboptimal birth outcomes (pre-term birth and/or a child being born SGA), stunting in a previous age-band, suboptimal feeding practices (age-appropriate breastfeeding and complementary foods), and incidence of diarrhoea (Figure A.**2**).
- Stunting increases the risk of mortality for children who have diarrhoea, pneumonia, measles and other illnesses.
- Odds ratios and relative risks are model inputs, can be changed, and have defaults based on the literature.

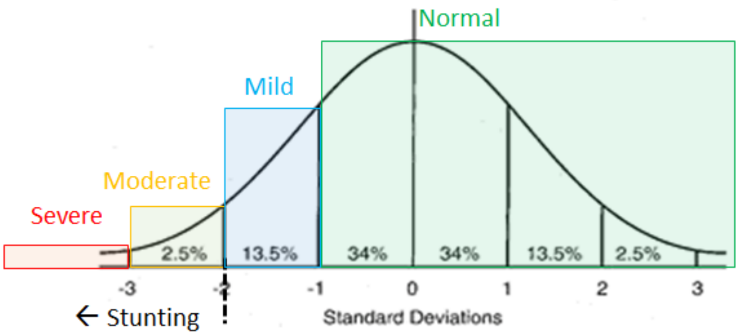

Figure A.3: The model divides children in each age-band into four height-for-age categories, based on WHO criteria. Children in the two lowest categories (severe and moderate) are considered to be stunted.

A.3 Modelling wasting using Optima Nutrition

- The weight-for-height distribution is tracked for children in each age band (Figure A.**4**, just like for stunting). Children are divided into four categories:
  - Severe acute malnutrition (SAM): < -3 standard deviations below than the median weight-for-height of the WHO reference population
  - Moderate acute malnutrition (MAM): < -2 and >= -3 standard deviations below than the median weight-for-height of the WHO reference population
  - Mild acute malnutrition: < -1 and >= -2 standard deviations below than the median weight-for-height of the WHO reference population
  - Normal: >= -1 standard deviation below than the median weight-for-height of the WHO reference population
- Children are considered to be "wasted" if they are in the SAM or MAM categories.
- Wasting is modelled as an incident (short-duration) condition:
  - As opposed to stunting, where being stunted in one age band increases the risk of being stunted in the next, wasting distributions are independent in each age band – this means that the distribution (i.e. prevalence) of wasting in a given time period does not affect the distribution of wasting in subsequent periods.
- Wasting increases the risk for mortality for children who have diarrhoea, pneumonia, measles and other illnesses
- Diarrhoea incidence and birth outcomes are risk factors for wasting (Figure A.**2**):
  - Reducing diarrhoea incidence can lead to reductions in wasting
  - Improvements in birth outcomes (term/pre-tern; appropriate for gestational age [AGA]/SGA) can lead to reduced wasting

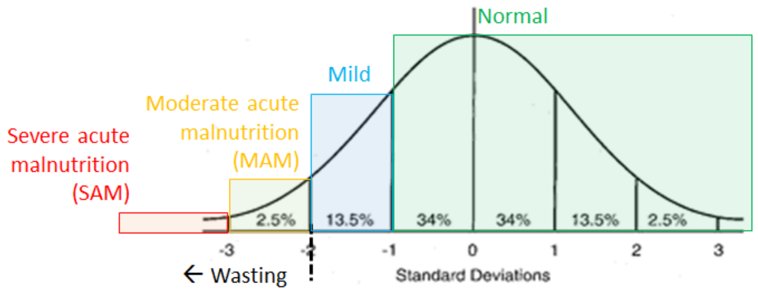

Figure A.4: The model divides children in each age-band into four weight-for-height Z-score (WHZ) categories. Children in the two lowest categories (SAM and MAM) are considered to be wasted.

Figure A.**5** shows the dynamics of the wasting model within each age band:

- Children enter the age band (shown from the left), and will be classified as SAM, MAM, mild or normal according to the prevalence of these states from the data
- Children in the mild and normal categories can develop MAM (incidence of MAM)
- Children with MAM can deteriorate to SAM (incidence of SAM)
- When they are in MAM or SAM categories, children have increased risks of death

- Children can recover from the SAM and MAM categories due to treatment.
- The incidence rates, probabilities of death and average duration spent in SAM and MAM are calibrated to match country-specific data on prevalence, mortality and treatment numbers.

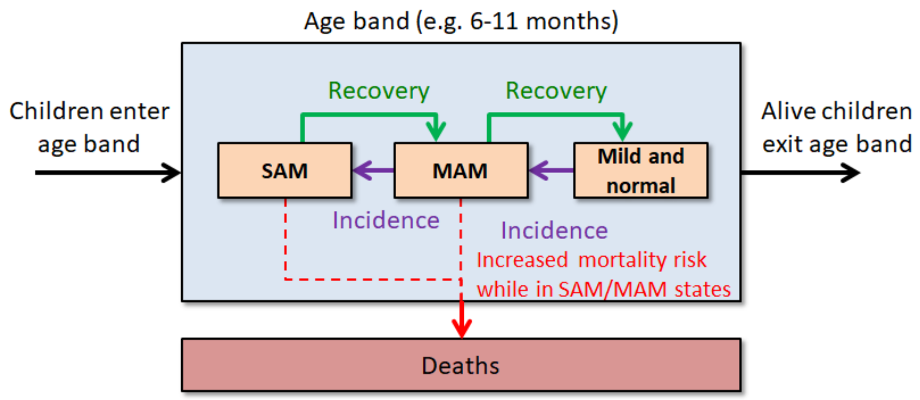

Figure A.5: Wasting is considered to be an acute condition within the model. The model tracks the incidence of severe acute malnutrition (SAM) and moderate acute malnutrition (MAM) within each age band, allowing children to cycle between categories.

A.4 Modelling anaemia using Optima Nutrition

Each population in the model is stratified by anaemia status: anaemic (mild, moderate, or severe) or not anaemic (Figure A.6). The model also includes a setting-specific input for the fraction of anaemia that is severe.

- Anaemia in pregnant women is modelled as a risk factor for maternal mortality (e.g. due to risk of haemorrhage)
- Anaemia in pregnant women is also modelled to be a risk factor for suboptimal birth outcomes
  - This can affect stunting, which in turn can affect mortality in children.

Non-pregnant women of Reproductive Age (WRA)	15 - 19 years	Not anaemic	Anaemic
	20 - 24 years	Not anaemic	Anaemic
	25 - 29 years	Not anaemic	Anaemic
	30 - 39 years	Not anaemic	Anaemic
	40 - 49 years	Not anaemic	Anaemic
Pregnant women	15 - 19 years	Not anaemic	Anaemic
	20 - 29 years	Not anaemic	Anaemic
	30 - 39 years	Not anaemic	Anaemic
	40 - 49 years	Not anaemic	Anaemic
Children<br>Also stratified by:<br>• Stunting<br>• Wasting<br>• Breastfeeding	0 - 1 months	Not anaemic	Anaemic
	1 - 6 months	Not anaemic	Anaemic
	6 - 11 months	Not anaemic	Anaemic
	12 - 23 months	Not anaemic	Anaemic
	24 – 59 months	Not anaemic	Anaemic

Figure A.6: Summary of model populations. Each population is stratified by anaemia status (anaemic or not anaemic)

APPENDIX B. Epidemiological and demographic input parameters

Table B.1: **Population and epidemiological data inputs.** Population-weighted average across 129 countries, as well as the median, inter-quartile range and range between countries. Model inputs for population size, mortality rates and stunting, wasting and anaemia prevalence are time-varying; values in table represent summaries of 2018 country estimates unless noted otherwise.

Parameter	*Global average*	*[Median; inter-quartile range (IQR) and range between countries]* *Low income country (LC), lower-middle income country (LMC) and upper-middle income country (UMC) averages*	Notes and sources
Population data			
Under-five population (2017)	Global total=595,148,000	[Between countries: median=1,193,852; IQR=277,206-3,682,272; range=8,183-119,756,815]<br>LC total=113,749,445, LMC total=310,761,347, UMC total=170,637,604	The World Bank Group Population Estimates and Projections[32], 2017
Percentage of population below the poverty line[#]	28.8%	[Between countries: median=24.3%; IQR=13.2-41.2%; range=0.4-82.3%]<br>LC average=44.3%, LMC average=28.1%, UMC average=19.4%	The World Bank Group Development Indicators[33] supplemented by regional estimates from LiST[34]
Percentage of population at risk of malaria	26.6%	[Between countries: median=0%; IQR=0-48.8%; range=0-100%]<br>LC average=60.6%, LMC average=22.3%, UMC average=7.9%	LiST[34]. Note: Default data used by LiST is from Guerra et al. 2008[35]
Mortality			The World Bank Group Development Indicators[33]
Neonatal mortality (per 1,000 live births)	17.1	[Between countries: median=14.9; IQR=8.6-24.2; range=1.5-44.2]<br>LC average=26.5, LMC average=17.7, UMC average=10.4	
Infant mortality (per 1,000 live births)	29.5	[Between countries: median=25.2; IQR=13.2-41.8; range=2.8-87.6]<br>LC average=48.7, LMC average=29.8, UMC average=16.6	
Under 5 mortality (per 1,000 live births)	39.6	[Between countries: median=30.4; IQR=15.2-58.5; range=3.5-127.2]<br>LC average=70.2, LMC average=40.1, UMC average=19	
Birth outcome distribution			Lee et al. 2013[36]
Pre-term SGA	2.2%	[Between countries: median=2.1%; IQR=1.9-2.5%; range=1-3.5%]<br>LC average=2.2%, LMC average=2.3%, UMC average=2%	
Pre-term AGA	9.1%	[Between countries: median=8.9%; IQR=7.5-10.5%; range=4-15.2%]<br>LC average=10.4%, LMC average=9.3%, UMC average=8%	
Term SGA	18.9%	[Between countries: median=16.6%; IQR=13.2-22.3%; range=2.5-44.5%]<br>LC average=26%, LMC average=19.8%, UMC average=13.3%	
Breastfeeding behaviours			

0-1 month			DHS[37], supplemented by regional estimates from LiST[34] for countries without DHS.<br>Note: LiST states "Coverage data for this indicator are drawn from DHS, MICS, and other nationally representative household surveys; however, they have been recalculated from the raw data sources to reflect the entire age period of interest."
Exclusive	43.3%	[Between countries: median=45.7%; IQR=28-61.3%; range=0.3-93.5%]<br>LC average=49.4%, LMC average=43.9%, UMC average=38.8%	
Predominant	20.5%	[Between countries: median=15.9%; IQR=11.7-26.9%; range=0-80.9%]<br>LC average=24.9%, LMC average=18.3%, UMC average=19.7%	
Partial	19.8%	[Between countries: median=18.2%; IQR=11.1-24.2%; range=0.4-72.4%]<br>LC average=14.8%, LMC average=20.4%, UMC average=22.5%	
None	16.2%	[Between countries: median=10%; IQR=3.3-24.3%; range=0-70.3%]<br>LC average=10.9%, LMC average=17.2%, UMC average=19%	
1-5 month			
Exclusive	33.7%	[Between countries: median=32.7%; IQR=20.2-44.6%; range=0.3-86.2%]<br>LC average=40.3%, LMC average=36.5%, UMC average=26.7%	
Predominant	23.6%	[Between countries: median=19.3%; IQR=14.2-29.6%; range=4.8-70.8%]<br>LC average=30.6%, LMC average=21.3%, UMC average=21%	
Partial	26%	[Between countries: median=24.1%; IQR=14.1-33.6%; range=4.8-73.4%]<br>LC average=20.8%, LMC average=26%, UMC average=29.5%	
None	17%	[Between countries: median=12.2%; IQR=5.1-25.7%; range=0.4-71.7%]<br>LC average=8.3%, LMC average=16.2%, UMC average=22.8%	
6-11 month			
Partial	66.1%	[Between countries: median=68.1%; IQR=49.1-89%; range=16-99%]<br>LC average=72.9%, LMC average=68.1%, UMC average=59.9%	
None	33.8%	[Between countries: median=31.9%; IQR=11-50.9%; range=1-84%]<br>LC average=27.1%, LMC average=31.9%, UMC average=40.1%	
12-23 months			
Partial	63%	[Between countries: median=69.6%; IQR=47.8-77%; range=11.7-94.5%]<br>LC average=75.2%, LMC average=67.6%, UMC average=50.8%	
None	37.4%	[Between countries: median=30.5%; IQR=23-52.2%; range=5.5-88.3%]<br>LC average=24.8%, LMC average=32.4%, UMC average=49.2%	
**Stunting prevalence**			
0-1 month			UN-JME[38], supplemented by DHS[37] where data were unavailable. For countries where stunting
Moderate (HAZ-score between -3 and -2)	7.4%	[Between countries: median=7.2%; IQR=5.1-9.6%; range=0-22.5%]<br>LC average=9%, LMC average=8%, UMC average=5.8%	
Severe (HAZ-score < -3)			

	5.0%	[Between countries: median=4.2%; IQR=2.6-6.7%; range=0-20.6%]<br>LC average=6%, LMC average=5.6%, UMC average=3.7%	prevalence was estimated to decrease across age brackets, we implemented a non-decreasing rule to enable it to be modelled.
1-5 month<br>*Moderate*<br>*Severe*	7.4%<br>5.0%	[Between countries: median=7.2%; IQR=5.1-9.6%; range=0-22.5%]<br>LC average=9%, LMC average=8%, UMC average=5.8%<br>[Between countries: median=4.2%; IQR=2.6-6.7%; range=0-20.6%]<br>LC average=6%, LMC average=5.6%, UMC average=3.7%	
6-11 month<br>*Moderate*<br>*Severe*	9.3%<br>6.2%	[Between countries: median=8.3%; IQR=6.2-12%; range=1.1-27.5%]<br>LC average=12.1%, LMC average=10%, UMC average=6.8%<br>[Between countries: median=5.4%; IQR=3.6-7.9%; range=0.5-20.6%]<br>LC average=8.1%, LMC average=6.7%, UMC average=4.5%	
12-23 months<br>*Moderate*<br>*Severe*	15.2%<br>10.4%	[Between countries: median=14.7%; IQR=8.3-21.4%; range=1.8-35.2%]<br>LC average=20.5%, LMC average=16.9%, UMC average=10%<br>[Between countries: median=8.6%; IQR=5.1-14.6%; range=0.9-32.9%]<br>LC average=14.3%, LMC average=12%, UMC average=6.3%	
24-59 months<br>*Moderate*<br>*Severe*	16.4%<br>11.7%	[Between countries: median=16.1%; IQR=10-22.7%; range=1.9-35.2%]<br>LC average=21.8%, LMC average=18.8%, UMC average=10.5%<br>[Between countries: median=10.1%; IQR=5.4-17%; range=0.9-32.9%]<br>LC average=17.2%, LMC average=13.4%, UMC average=6.6%	
Wasting prevalence			
0-1 month<br>*Moderate (WHZ-score between -3 and -2)*<br>*Severe (WHZ-score < -3)*	5.5%<br>3.6%	[Between countries: median=4.6%; IQR=3-7.6%; range=0.5-16.7%]<br>LC average=6.2%, LMC average=6.1%, UMC average=4.5%<br>[Between countries: median=2.6%; IQR=1.6-5.3%; range=0-14.1%]<br>LC average=4.4%, LMC average=4.1%, UMC average=2.7%	UN-JME[38], supplemented by DHS[37] where data were unavailable.
1-5 month<br>*Moderate*<br>*Severe*	5.5%<br>3.6%	[Between countries: median=4.6%; IQR=3-7.6%; range=0.5-16.7%]<br>LC average=6.2%, LMC average=6.1%, UMC average=4.5%<br>[Between countries: median=2.6%; IQR=1.6-5.3%; range=0-14.1%]	

		LC average=4.4%, LMC average=4.1%, UMC average=2.7%	
<u>6-11 month</u><br>*Moderate*<br>*Severe*	6.0%<br>2.8%	[Between countries: median=5.5%; IQR=2.3-8.8%; range=0.2-16.2%]<br>LC average=8.7%, LMC average=6.8%, UMC average=3.5%<br>[Between countries: median=2%; IQR=1.1-4%; range=0-13.2%]<br>LC average=3.9%, LMC average=3.4%, UMC average=1.6%	
<u>12-23 months</u><br>*Moderate*<br>*Severe*	4.9%<br>2.2%	[Between countries: median=3.8%; IQR=1.8-6.9%; range=0.1-15.7%]<br>LC average=7.3%, LMC average=5.9%, UMC average=2.5%<br>[Between countries: median=1.7%; IQR=0.7-3.2%; range=0-12.4%]<br>LC average=3.2%, LMC average=2.7%, UMC average=1.1%	
<u>24-59 months</u><br>*Moderate*<br>*Severe*	3.8%<br>1.5%	[Between countries: median=2.9%; IQR=1.4-4.9%; range=0.2-15.7%]<br>LC average=4.5%, LMC average=4.4%, UMC average=2.6%<br>[Between countries: median=1%; IQR=0.5-2.1%; range=0-11.6%]<br>LC average=2%, LMC average=1.8%, UMC average=1%	
Anaemia prevalence			
Women of reproductive age	29.2%	[Between countries: median=27.5%; IQR=20.2-36.7%; range=6.9-71.8%]<br>LC average=36%, LMC average=27.7%, UMC average=22%	Global Burden of Disease Study 2017[39]
Pregnant women	39.6%	[Between countries: median=37.2%; IQR=29.2-48.6%; range=10.7-79.1%]<br>LC average=44.4%, LMC average=35.7%, UMC average=33%	DHS[37], supplemented by regional estimates from LiST[34] for countries without DHS.<br>Note: Default data used by LiST is from Stevens et al. 2013[40]
Diarrhoea incidence (under-five; average episodes per year)	2.0	[Between countries: median=2; IQR=1.4-2.5; range=0.4-4.7]<br>LC average=2.5, LMC average=2, UMC average=1.7	Walker et al. 2013[41]

Using national poverty lines.

Abbreviations: AGA, appropriate for gestational age; DHS, Demographic and Health Survey; HAZ-score, height-for-age Z score; IQR, inter-quartile range; LC, low income country; LiST, Lives Saved Tool; LMC, lower-middle income country; MICS, Multiple Indicator Cluster Survey; SGA, small for gestational age; UMC, upper-middle income country; UN-JME, United Nations Joint Malnutrition Estimates; WHZ-score, weight-for-height Z-score.

APPENDIX C. Other model parameters

C.1 Mortality risk factors

C.1.1 Birth outcomes

Table C.1: Relative risk ratios for neonatal mortality types by birth outcome (term / pre-term and appropriate for gestational age [AGA] / small for gestational age [SGA])

Birth outcome	Neonatal sepsis	Neonatal pneumonia	Neonatal asphyxia	Neonatal prematurity
Term AGA	Ref	Ref	Ref	Ref
Term SGA	2.07	2.07	2.07	1
Pre-term AGA	8.02	8.02	8.02	999.99
Pre-term SGA	11.54	11.54	11.54	999.99

Source: Katz et al. 2013 [42]

C.1.2 Stunting

Table C.2: Relative risk ratios for 1-59 month old mortality types, by height-for-age Z-score (HAZ) category

Age group	HAZ-status	Diarrhoea	Pneumonia	Meningitis	Measles	Other
1-59 months	None (HAZ-score >=-1)	Ref	Ref	Ref	Ref	Ref
	Mild (HAZ-score >= -2 and < -1)	1.67	1.55	1	1	1
	Moderate (HAZ-score >= -3 and < -2)	2.38	2.18	1.86	2.79	1.86
	Severe (HAZ-score < -3)	6.33	6.39	3.01	6.01	3.01

Source: Olofin et al. 2013 [43]

C.1.3 Wasting

Table C.3: Relative risk ratios for 1-59 month old mortality types, by weight-for-height Z-score (WHZ) category

Age group	Status	Diarrhoea	Pneumonia	Meningitis	Measles	Other
1-59 months	None (WHZ-score >= -1)	Ref	Ref	Ref	Ref	Ref
	Mild (WHZ-score >= -2 and < -1)	1.6	1.92	1.65	1	1.65
	MAM (WHZ-score >= -3 and < -2)	3.41	4.66	2.73	2.58	2.73
	SAM (WHZ-score < -3)	12.33	9.68	11.21	9.63	11.21

Source: Olofin et al. 2013 [43]

C.1.4 *Breastfeeding practices*

Table C.4: Relative risk ratios for mortality types, by breastfeeding practices and age group

Age group	Status	Neonatal diarrhoea / sepsis / pneumonia*	Diarrhoea†	Pneumonia^	Meningitis / measles / pertussis^
<1 month	Exclusive	Ref			
	Predominant	1.35			
	Partial	1.35			
	None	5.40			
1-5 months	Exclusive		Ref	Ref	
	Predominant		2.28	1.66	1.48
	Partial		4.62	2.50	2.84
	None		10.53	14.97	14.40
6-11 months	Partial		Ref	Ref	Ref
	None		1.47	1.92	3.69
12-23 months	Partial		Ref	Ref	Ref
	None		2.57	1.92	3.69

Sources: * NEOVITA Study Group 2016 [44] with predominant / partial assuming late initiation; † Lamberti et al. 2011 [45]; ^ Lamberti et al. 2013 [46].

C.1.5 *Anaemia*

Table C.5: Relative risks of maternal mortality types by anaemia status.

Pregnant women age in years	Status	Antepartum haemorrhage	Intrapartum haemorrhage	Postpartum haemorrhage
15-49	Not anaemic	Ref	Ref	Ref
15-49	anaemic	10.675	10.675	10.675

Source: LiST [34].
Applies only to the fraction who are severely anaemic

C.2 Birth outcomes

C.2.1 *Impact of birth outcomes on stunting, wasting and anaemia*

Table C.6: Odds ratios for stunting (HAZ-score <-2) and wasting (WHZ-score <-2), by birth outcome

	Odds ratio for condition if born:			
Condition	Term AGA	Term SGA	Pre-term AGA	Pre-term SGA
Stunting	Ref	5	6.4	46.5
Wasting	Ref	2.52	1.96	4.19

Sources: stunting LiST [34]; wasting Christian et al. 2013 [47] for low and middle income countries.

C.2.2 Odds of birth outcomes with maternal anaemia

Table C.7: Odds ratios for being born term / pre-term and appropriate for gestational age [AGA] / small for gestational age [SGA] if mother is anaemic

Condition	Term SGA^	Pre-term AGA†	Pre-term SGA^
No maternal anaemia	Ref	Ref	Ref
Maternal anaemia	1.53	1.32	1.53

Sources: ^ Child Health Epidemiology Reference Group 2011 [48]; † Xiong et al. 2015 [49].

C.3 Impact of diarrhoea on stunting, wasting and anaemia

Table C.8: Odds ratios for stunting, wasting and anaemia as diarrhoea incidence increases.

Condition	Age band	Odds ratio for every additional episode	Source
Stunting	0-59 months	1.025	LiST [34]
Wasting	0-59 months	1.025	Assumed the same as stunting

C.4 Impact of past stunting on stunting

Table C.9: Odds ratios for continued stunting (<-2 HAZ-score) if stunted in a prior age band

Age in months	Odds Ratio
1-5	45
6-11	361.6
12-23	174.7
24-59	174.7

Source: LiST [34].
